# Climate Change Favors African Malaria Vector Mosquitoes

**DOI:** 10.1111/gcb.70610

**Published:** 2025-11-26

**Authors:** Tiem van der Deure, David Nogués‐Bravo, Lembris Laanyuni Njotto, Anna‐Sofie Stensgaard

**Affiliations:** ^1^ Department for Veterinary and Animal Sciences, Section for Parasitology and Pathobiology University of Copenhagen Copenhagen Denmark; ^2^ Center for Macroecology, Evolution and Climate, Globe Institute University of Copenhagen Copenhagen Denmark; ^3^ College of Information and Communication Technologies University of Dar Es Salaam Dar Es Salaam Tanzania; ^4^ Department of Mathematics and ICT College of Business Education Dar Es Salaam Tanzania

**Keywords:** *Anopheles*, climate change, disease vector, malaria, mosquito‐borne disease, species distribution model

## Abstract

Malaria, a parasitic disease transmitted by mosquitoes of the genus *Anopheles*, causes half a million deaths annually, mostly among children in Africa. While climate change is expected to significantly alter malaria transmission, previous forecasts have largely overlooked the species‐specific responses of mosquito vectors which may substantially impact the outcome of such forecasts. Here, we for the first time estimate the future human exposure to each of six dominant African malaria vector species. Using an extensive mosquito observation dataset, robust species distribution modeling, and climate and land‐use data, we investigate the climatic niches of six dominant African malaria vector species and map out their differing responses to climate and land use change across sub‐Saharan Africa. Projections of future vector suitability identify three species that are likely to experience a substantial expansion of suitable habitat: *Anopheles gambiae* , *Anopheles coluzzii*, and *Anopheles nili* s.l. By combining these projections with human population density data, we conservatively estimate that approximately 200 million additional people could be living in areas highly suitable for these three vector species by the end of the century, with new hotspots of human exposure emerging in Central and East Africa. Our results align with observed historical range shifts of *Anopheles* species but stand in contrast to earlier studies that have predicted climate change would have little effect on or even reduce malaria transmission. We find that climate change impacts on malaria vectors are highly species‐specific, emphasizing the need for longitudinal field studies and integrated modeling approaches to address the ongoing redistribution of malaria vectors. As the world strives for malaria elimination amidst accelerating climate change, our study underscores the urgent need to adapt malaria control strategies to shifting vector distributions driven by environmental change.

## Graphical abstract

Malaria is a deadly parasitic disease that is transmitted by mosquitoes. This study investigates occurrence patterns of malaria mosquito species to predict how climate change will affect their range in sub‐Saharan African, where most malaria deaths occur. It highlights three mosquito species that could expand their range throughout Africa because of climate change, potentially affecting hundreds of millions of people.

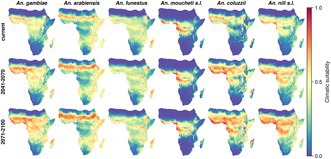

## 1 Introduction

Malaria is a mosquito‐borne parasitic disease with around 250 million cases annually. It causes approximately half a million deaths, more than 90% of which occur in sub‐Saharan Africa (Weiss et al. 2019; World Health Organization 2024). Malaria transmission is heavily influenced by environmental factors. Warm temperatures accelerate the development of *Plasmodium*, the protozoan parasite that causes malaria, within the mosquito, making transmission more efficient, while rainfall creates breeding sites for mosquitoes, and humidity and temperature affect mosquito survival (Mordecai et al. 2013). As the planet warms, understanding possible shifts in the distribution and burden of malaria as a consequence is crucial for the continued effectiveness of control and eradication efforts (Nkumama et al. 2017; Ryan et al. 2020). However, despite years of research, the historical and possible future impacts of climate change on malaria have been difficult to resolve (Béguin et al. 2011; Carlson et al. 2024; Klepac et al. 2024).

To date, most research on the effects of climate change on malaria has focused on the temperature dependence of the transmission efficiency of malaria parasites, typically through mechanistic models parameterized on experimental life history data (Beloconi et al. 2023; Eckhoff 2011; Mandal et al. 2011; Suh et al. 2024). A number of studies suggest climate change could put new human populations at risk in cooler areas (M'Bra et al. 2018; Mordecai et al. 2020; Ngarakana‐Gwasira et al. 2016; Semakula et al. 2017), in particular in East Africa (Bouma et al. 2016). In hotter areas such as West Africa and the Sahel, previous modeling has predicted that climate change would not have much impact or even lead to a decline in malaria transmission (Caminade et al. 2014; Carlson et al. 2024; Colón‐González et al. 2021; Yamana et al. 2016).

While these studies have significantly improved our understanding of how temperature affects the malaria parasite's (of which the most important in an African context is *Plasmodium falciparum*) life cycle, they only to a limited extent account for the complex ecology and biology of the involved mosquito vectors. Most models focus on a single vector species (usually the archetypical vector *Anopheles gambiae* ) (Smith et al. 2018), despite the fact that malaria transmission in sub‐Saharan Africa involves around 10 dominant and over a dozen secondary *Anopheles* vector species (Kyalo et al. 2017; Sinka et al. 2010), each with their unique behaviors, environmental tolerances, and habitat preferences (Sinka et al. 2010). For instance, *An. arabiensis* is an outdoor‐biting species associated with dry areas and temporary pools, whereas *An. funestus* breeds in larger, permanent larval habitats and tends to bite indoors (Nzioki et al. 2023). The diversity of malaria vectors is likely to play a key role in the persistence of malaria transmission, as well as in future transmission patterns. Because of their differing behaviors and ecologies, secondary vectors can elude control measures and sustain residual transmission in response to malaria control efforts (Sherrard‐Smith et al. 2019). Environmental (e.g., drought (Kent et al. 2007)) or human (e.g., forest clearance (Manga et al. 1995)) disruption has for instance been shown to trigger a shift from one malaria vector to another. Potentially, climate change could result in shifts in the relative importance of malaria vectors for malaria transmission, rather than the cessation of transmission.

A growing field of literature is available on the distribution of malaria vectors, including extensive, freely available databases of mosquito observations, as well as distribution maps for nine malaria vector species (Irish et al. 2020; Kyalo et al. 2017; Sinka et al. 2010; Wiebe et al. 2017). How each of these vectors will respond to climatic changes will have major implications for malaria transmission, but surprisingly few studies have investigated the potential consequences for human health of range shifts of African malaria vectors (Lippi et al. 2023). Earlier studies have either modeled just one or two mosquito species (*An. gambiae* and/or *An. arabiensis*) (Drake and Beier 2014; Peterson 2009; Tonnang et al. 2010, 2014), have forecast the changing availability of breeding sites without considering the different ecological requirements of individual species (Smith et al. 2020, 2024), or have investigated the changing species richness of *Anopheles* species without making a distinction between the importance of these species as malaria vectors (Carlson et al. 2023; Nie et al. 2024). While earlier studies have investigated future exposure to the invasive vector *Anopheles stephensi* (Acosta et al. 2025), as well as to vectors in southern Europe (Hertig 2019) and South America (Laporta et al. 2015) in detail, no study to date has estimated the future human exposure to African malaria vectors other than *An. gambiae* and *An. arabiensis*. Therefore, we still have a limited understanding of how range shifts of these vector species might affect human health.

To address these gaps, we examine how climate and land use shape the current and future distributions of six dominant malaria vector species in sub‐Saharan Africa. First, we model the environmental suitability of each species to assess their potential range shifts under future climate and land‐use scenarios. Second, we evaluate how well modelled suitability correlates with malaria prevalence to explore the relationship between vector environmental suitability and disease risk. Finally, we estimate future human exposure by identifying where increasing environmental suitability for these vectors is likely to overlap with high population densities, highlighting potential emerging hotspots of malaria risk. By combining ecological niche theory (Colwell and Rangel 2009; Holt 2009), species distribution models (SDMs), and machine learning methods (Ryo et al. 2021) with climate and land‐use scenarios, we leverage the currently most extensive databases on mosquito vector occurrences to project future distribution patterns of vector mosquitoes and assess how many people could be impacted by these range shifts and where. Understanding these shifts is crucial, as climate change presents a significant challenge to malaria control and eradication efforts (Fornace et al. 2021; Nissan et al. 2021). By pinpointing the malaria vector species that are favored by climate and land use change and the regions where these might emerge, this study provides critical insights to inform targeted vector control strategies, optimize resource allocation, and inform public health policies (Larsen et al. 2020).

## 2 Materials and Methods

### 2.1 Mosquito Occurrence Data

Based on earlier reviews, Sinka et al. propose seven dominant vector species in Sub‐Saharan Africa: *An. gambiae*, *An. arabiensis*, *An. funestus* s.s., *An. arabiensis*, *An. merus*, *An. melas*, *An. nili* s.l., and *An. moucheti* s.l. (Sinka et al. 2010). Of these, *An. merus* and *An. melas* are saltwater species restricted to coastal regions, and we therefore excluded them from this study. Furthermore, in 2013 the M‐form of *An. gambiae* was recognized as a separate species and has since been known as *An. coluzzii* (Coetzee et al. 2013). Both *An. gambiae* and *An. coluzzii* are highly efficient malaria vectors, with distinct behaviors and environmental preferences. We therefore model both separately. In total, we thus model six Anopheles species that all can act as primary malaria vectors: *An. gambiae*, *An. coluzzii*, *An. arabiensis*, *An. moucheti* s.l., *An. nili* s.l, and *An. funestus*.

Mosquito occurrence data was extracted from a database published by Kyalo et al. (2017), which is one of the most extensive databases on *Anopheles* species, including more than a century of records of 21 species (Kyalo et al. 2017; Snow 2017a), and the database collated by the Malaria Atlas Project, which was accessed both through a published dataset (Massey et al. 2016, 2017) and the malariaAtlas R package (Pfeffer et al. 2018).

**FIGURE 1:**
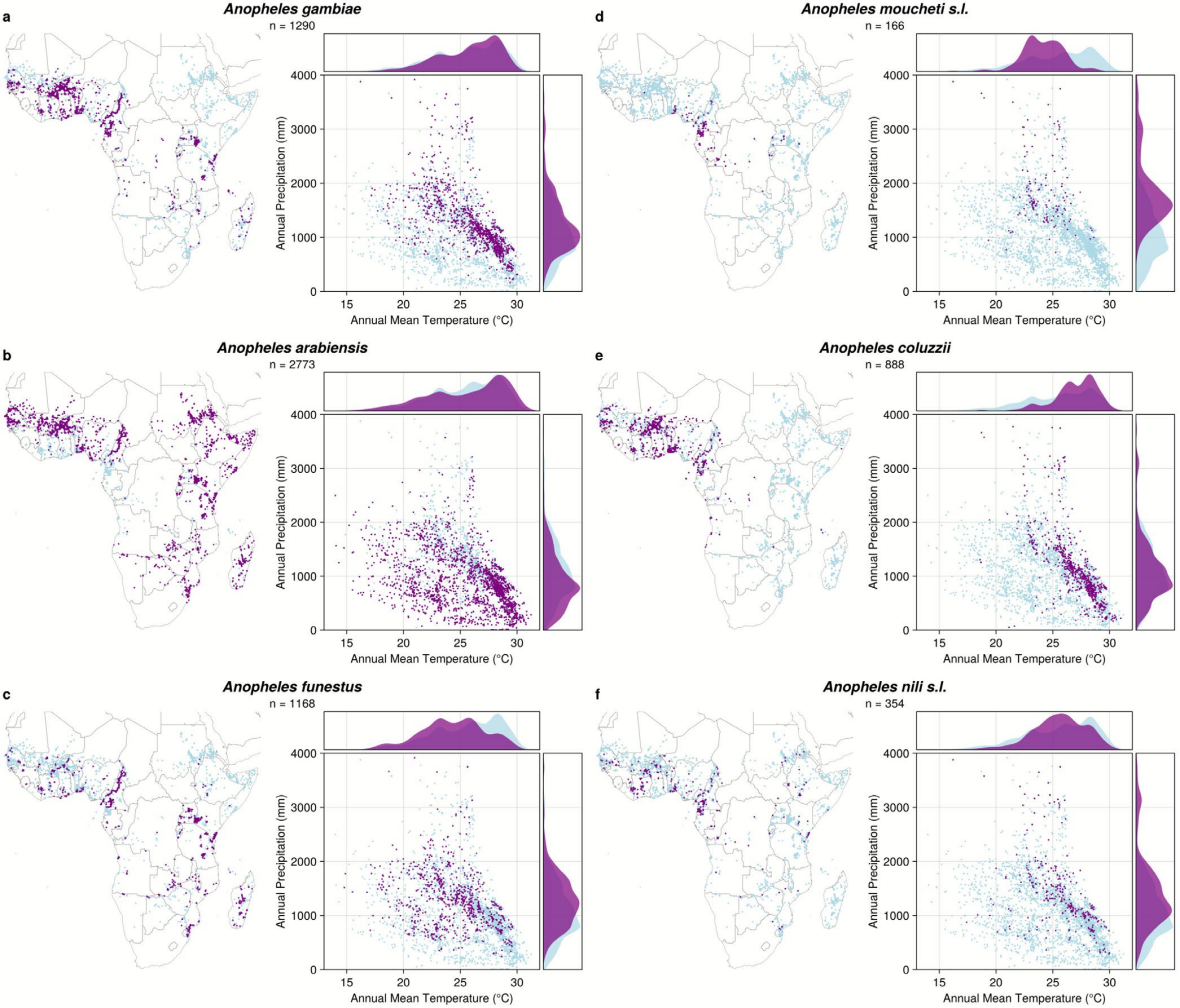
Occurrences records in geographic and climatic space for six important African malaria vectors: (a) *Anopheles gambiae*, (b) *Anopheles arabiensis*, (c) *Anopheles funestus*, (d) *Anopheles moucheti* s.l., (e) *Anopheles coluzzii*, and (f) *Anopheles nili* s.l. For each species, the geographic locations (maps on the left) and climatic conditions (scatter and density plots on the right) of occurrence points are shown in purple. For comparison, records of all *Anopheles* species are shown in light blue on each map and plot. The number of occurrence records for the species after data cleaning and thinning shown below each plot title. Map lines are downloaded from Natural Earth and do not necessarily depict accepted national boundaries.

The databases were collated, different synonymous species designations were converted as appropriate (e.g., *An. gambiae* M form to *An. coluzzii*) and duplicate records were removed. We filtered out data if collection took place entirely outside of the 1980 to 2010 period in order to match the period of the 30‐year average of the climate data. We used spatial thinning in order to reduce spatial bias, employing a thinning distance of 10 km (Boria et al. 2014). The number of records for each species after each filtering step is given in Table S1. This resulted in between 166 (for *An. moucheti* s.l.) and 2773 (for *An. arabiensis*) records for each species (Figure 1).

### 2.2 Background Data

Occurrence data often has spatial bias, resulting from unevenly distributed sampling efforts. It is challenging to determine whether spatial patterns are a result of sampling bias or differences in habitat suitability. Different strategies for generating background points range from sampling randomly in geographic space, sampling in environmental space, or using presence records of similar species as background points (Barber et al. 2022; Barbet‐Massin et al. 2012). Here, we choose a middle‐of‐the‐road approach, sampling 50% of background points randomly within the region of interest (as defined below), and 50% from sampling locations where other anopheline species were found. The total number of background points generated was twice the number of presences.

Since the species we modelled are all distributed throughout Sub‐Saharan Africa, but none of them are found north of the Sahara, we used the same region of interest for all investigated species and defined it based on the Sahara Desert. The northern boundary of the region of interest was defined by taking the boundary where precipitation is at 200 mm per year and buffering this by 7 degrees (approx. 750 km) (see Figure S1).

### 2.3 Climate and Land Use Data

We used climate data from CHELSA, which provides bias‐corrected climate variables with global coverage at a 1 km spatial resolution (Karger et al. 2017). For future projections, we used projections for the mid‐century (2041–2070) and end of the century (2071–2100), under a low (SSP1‐2.6) and a high (SSP3‐7.0) emissions scenario for each of the five global circulation models (GCMs) available through the CHELSA website. CHELSA data were aggregated to a resolution of 2.5 arcminutes (ca. 5 km).

In addition to climate data, we used a global land use product with 7 land cover features (Chen et al. 2022). This dataset is also available under the SSP1‐2.6 and SSP3‐7.0 scenario. For the mid‐century, the projection for 2055 was used and for the end of the century the projection for 2085. The data was resampled to match the resolution and projection of the climate data using mode resampling (taking the most commonly occurring land cover category). Any cells classified as Water were masked. As no grid cells had Snow and Ice cover, five features remained: Forest, Grassland, Cropland, Barren, and Urban.

### 2.4 Ensemble Modeling

Ensemble modeling involves fitting multiple machine learning models to the same dataset and combining their outputs. This approach is widely used in SDMs to mitigate algorithmic uncertainty (Araújo and New 2007). Here, we used an ensemble with three models: a generalized additive model (GAM), Maxnet (a close analogue to Maxent) (Phillips et al. 2017), and an artificial neural network (ANN). These models were selected for their ability to capture complex interactions between variables without being too prone to overfitting, and we tweaked model settings to further reduce the risk of overfitting. We limited the flexibility of the GAMs by setting the *k*‐parameter of the smooth term to 5 and the gamma parameter to 3. For Maxnet, the feature classes were limited to linear, quadratic, and product (excluding hinge and threshold features to limit model complexity (Merow et al. 2013)). For the ANNs, we used only a single hidden layer with 16 neurons and fit for 50 epochs.

Modeling was done in Julia v1.11, a high‐performance scientific computing programming language, using the MLJ machine learning framework (Bezanson et al. 2017). For GAMs, we used the *mgcv* library in R (Wood 2011), interfacing between the two programming languages with the Julia package RCall. We used Julia's Flux.jl package to fit ANNs (Innes 2018; Innes et al. 2018) and Maxnet.jl to fit Maxnet models.

Spatial autocorrelation can inflate performance metrics for models using spatial data, as has often been observed in SDMs (Hijmans 2012; Radosavljevic and Anderson 2014). Here, we use fivefold spatial cross‐validation to more accurately measure the performance of the ensemble model. The study region was divided into squares of 3 by 3 degrees (approx. 300 by 300 km), which were then randomly distributed into five folds. The ensemble was fit five times, leaving out one fold for evaluation each time. To assess the performance of the model, we used the Area under the Receiver Operating Characteristic Curve (AUC), the True Skill Statistic (TSS), and the Continuous Boyce Index (CBI) (Allouche et al. 2006; Hirzel et al. 2006). These metrics capture different aspects of model performance; whereas AUC and TSS measure the ability of the model to discriminate between presence and absence points, the CBI is a presence‐only metric that assesses if areas with higher suitability scores consistently contain more occurrence records. We report the average of each performance metric over the five folds.

We explored two strategies for the selection of bioclimatic variables for modeling. First, we used a forward variable selection procedure, iteratively adding the variable that improved AUC most to the set of predictors until no variable further improved performance. Second, we manually selected two temperature and three precipitation variables that we deemed biologically relevant for the vector species investigated and were not strongly correlated in the region of interest to avoid multicollinearity issues (Pearson correlation coefficient < 0.7 for all variables, see Figure S2). These were bio1 (Annual Mean Temperature), bio7 (Temperature Annual Range), bio12 (Annual Mean Precipitation), bio14 (Precipitation of Driest Month), and bio15 (Precipitation Seasonality). Both approaches resulted in very similar performances (Tables S2 and S3). To reduce the risk of data dredging or overfitting and make comparisons of variable contributions between species easier, we opted for the second strategy. For all further analysis, the aforementioned set of five bioclimatic variables and land use classes was used for model fitting and projection.

After model validation, ensembles were fit using all data available for each species. These ensembles were then used to generate projections to current and future climates. The final prediction is the simple mean of the prediction of each model in the ensemble. For predictions under future climate conditions, predictions were generated for each of five GCMs and then averaged.

### 2.5 Model Interpretations

We used Shapley values to understand how each variable contributes to the ensemble prediction and their relative importance. The Shapley value is a game theory concept that has been adopted to assess variable contributions in machine learning models (Lundberg and Lee 2017; Štrumbelj and Kononenko 2014), including SDMs (He et al. 2022; Ryo et al. 2021; Song and Estes 2023). The Shapley value quantifies the contribution of each variable to a prediction and can be obtained by averaging the change in outcome when adding all variables in all possible orders. Shapley values have several desirable features, such as effectively handling multicollinearity and synergistic effects (Roth 1988). The sum of Shapley values of all variables is equal to the difference between the expected outcome and model outcome.

To understand how each variable contributes to the suitability under current conditions, we use a Monte Carlo algorithm (Štrumbelj and Kononenko 2014). This algorithm works by substituting variables in each grid cell with variables from a random other grid cell in random order and observing the resulting change in suitability. We performed 32 Monte Carlo samples for each grid cell and each variable. Because Shapley values are additive (Roth 1988), we were able to calculate the ensemble Shapley value of any grid cell as the mean of the Shapley values of each model in the ensemble for that grid cell. The variable importance of each variable was calculated as the mean absolute Shapley value of the resulting grid.

Then, to understand which variables drive the change in suitability between current and future environmental conditions, we used a related algorithm called Baseline Shapley, where input variables are instead compared with some self‐defined baseline (Sundararajan and Najmi 2020). Here, we define current environmental conditions as the baseline and future conditions as the input and calculate the mean change in suitability for each variable as future conditions are substituted for the current conditions in every possible order.

### 2.6 Correlation With Malaria Prevalence

To investigate how malaria vector suitability is associated with malaria prevalence, we use a recently published compendium of over 50,000 malaria prevalence surveys collated by Snow (2017b). The dataset includes an age‐corrected childhood malaria rate (estimated *P. falciparum* positivity rate for children 2–10 years old) for each survey. For our analysis, we only consider surveys recorded between 1980 and 2010, matching the considered climate period. In addition, when multiple surveys were recorded within the same grid cell, we used the mean positivity rates of the surveys conducted. This resulted in childhood malaria estimates for 13,070 unique grid cells.

To visualize how *P. falciparum* prevalence changes with the suitability of each vector species, we assign each grid cell with survey data to one or more overlapping bins 0.2 suitability units wide and spaced 0.05 units apart. We then plot the median value and interquartile range of each bin. In addition, we calculate the Pearson's correlation coefficient between *P. falciparum* rate and the modelled suitability in each grid cell for each vector species. Because spatial auto‐correlation increases the uncertainty of correlation estimates (Legendre 1993; Lennon 2000), we here only report the correlation coefficient and refrain from making any significance claims.

### 2.7 Populations Living in Areas Highly Suitable for Malaria Vectors

To assess the number of people potentially impacted by the changes in the distribution of *Anopheles* species, we combined the mosquito suitability maps with global gridded 1 km population data for 2020 available from WorldPop (Lloyd et al. 2019; WorldPop 2018). Firstly, we identified areas with high suitability for each vector species using a cut‐off corresponding to the 10th percentile training presence (Radosavljevic and Anderson 2014). This cut‐off was chosen to make it easier to compare estimates between species, and to exclude areas with few occurrence records. We estimate the number of people living in areas with a highly suitable climate for a particular vector species by taking the sum of the population counts of all grid cells where the suitability was above this cut‐off. For future predictions, we calculate this for each GCM and then calculate the mean and standard deviation across estimates.

We used two approaches for future projections of human population counts. In our central estimate, we use a conservative approach and assume no change in population counts to isolate the effects of climate change. To get an alternative estimate of the number of people exposed under each socio‐economic scenario, we adjust for future demographic changes by multiplying gridded population counts with the forecasted demographic change by country for 2055 and 2085, relative to 2020. We use population forecasts from global SSP‐specific population projections (KC et al. 2024). This approach provides grids that are specific to socio‐economic scenarios and consistent with both the original gridded population counts and country‐level changes, but does not account for changes within countries, such as urbanization.

## 3 Results

### 3.1 Different Climatic Preferences of Malaria Vectors

To contextualize model predictions and understand differences in the realized climatic niches of each of the six dominant malaria vector species, we first visually analyzed climatic conditions at their occurrence locations (Soberón 2007). This analysis shows that the investigated mosquito species have markedly different climatic niches (Figure 1). For instance, *An. arabiensis* and *An. funestus* (Figure 1b,c) are climate generalists with wide climatic niches, while *An. coluzzii* and *An. moucheti* s.l. (Figure 1d,e) are constrained to a narrower range of climatic conditions.

The realized niches of each species are defined by different temperature and precipitation thresholds. *An. gambiae*, *An. arabiensis*, and *An. coluzzii* are often found in areas with annual mean temperatures of 25°C or above, but while *An. arabiensis* is recorded in extremely dry areas, *An. gambiae* is rarely found in areas with less than 500 mm precipitation per year (Figure 1). Geographically, *An. arabiensis* is also very widely spread, extending north into the Sahara Desert and south into South Africa. Most records for *An. nili* s.l. have high precipitation, but more moderate temperatures than for *An. gambiae*. *An. moucheti* s.l. is the most specialized of all investigated species and is exclusively recorded in tropical areas in Central Africa, with intermediate temperatures (around 25°C) and more than 1000 mm annual precipitation. Finally, *An. funestus* is relatively often found in East Africa, including in more temperate areas with intermediate temperatures and precipitation.

### 3.2 Projected Shifts in Environmental Suitability of Key Malaria Vectors

**FIGURE 2:**
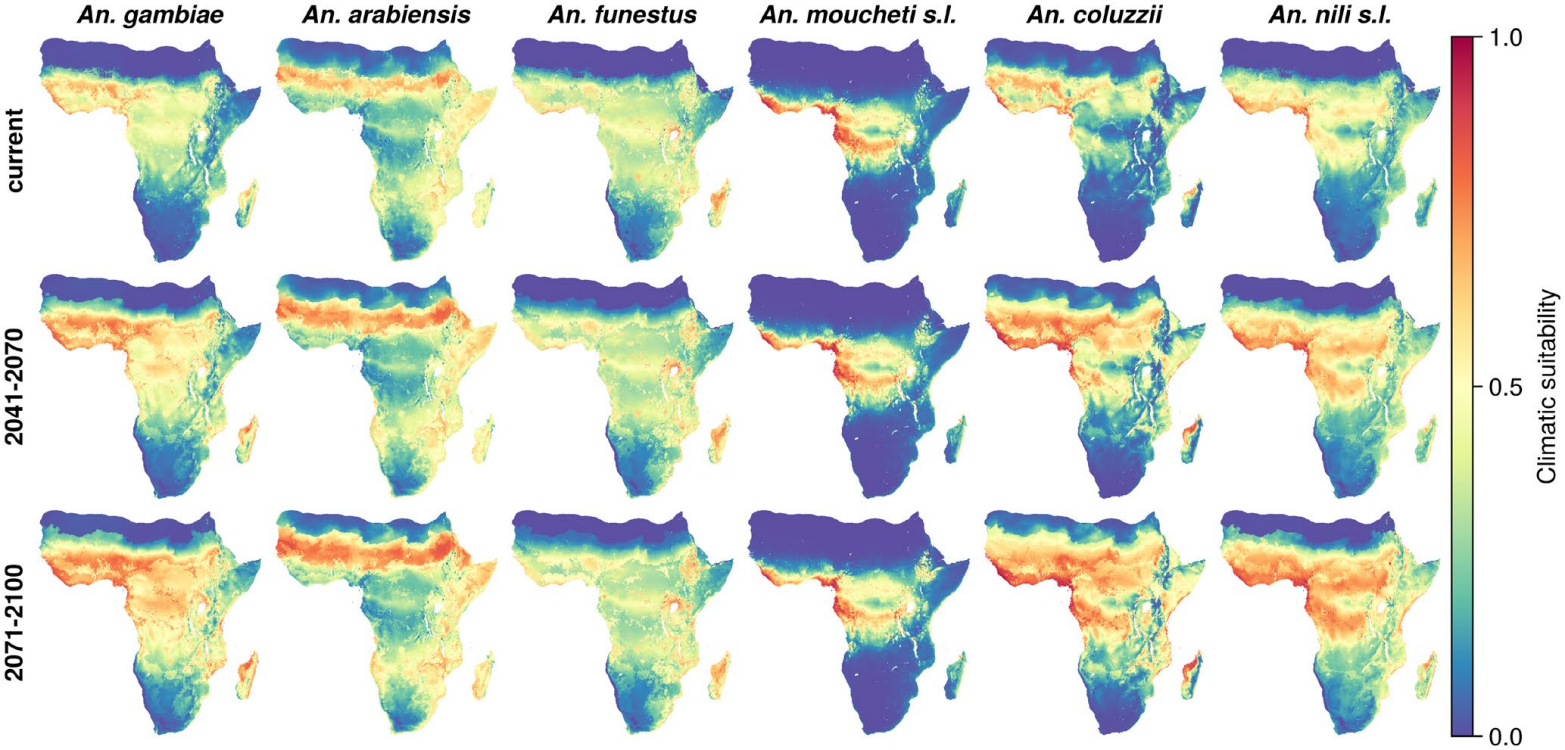
Climatic suitability of African malaria vector species under current (top row) and future (middle and bottom row) conditions. Climate and land use projections shown are under the SSP3‐7.0 scenario with higher emissions. Climatic suitability is projected to increase significantly for *An. gambiae*, *An. coluzzii*, and *An. nili* s.l., whereas it remains stable for *An. arabiensis*, *An. funestus*, and *An. moucheti* s.l.

To investigate the current and future environmental suitability of the six species, we fitted ensemble models using land use and climate data as covariates. Our future projections show substantial increases in environmental suitability under future climatic and land use conditions for *An. gambiae, An. coluzzii*, and *An. nili* s.l., and minimal variation for the other three species (Figure 2).

Suitable areas for *An. gambiae* are currently in West Africa, around the Great Lakes and in Mozambique whereas suitable areas for *An. coluzzii* are concentrated in West Africa and for *An. nili* s.l. in West and tropical Central Africa. For all these three species, suitability is projected to rise in already suitable areas, such as West Africa, and expand into currently less suitable regions, including East Africa. This upward trend persists throughout the century, showing clear progression from current conditions to mid‐century and end‐of‐century scenarios. Under the SSP1‐2.6 scenario, which assumes far‐reaching action to mitigate climate change, the projected increases in environmental suitability are much smaller (Figure S3).

For *An. arabiensis* and *An. funestus*, which we found are climate generalists, most of Sub‐Saharan Africa is climatically suitable, except for regions in South Africa and Central Africa in the case of *An. arabiensis*. The suitable area for *An. moucheti* s.l. is restricted to tropical Central Africa (Figure 2, top row). We project that the suitable areas for these three species will remain the same under climate change (Figure 2, middle and bottom row).

The model performance of the ensembles, measured by spatially cross‐validated AUC, TSS, and CBI was adequate for all species (Table S3). The models for the different species had AUC values between 0.72 and 0.84, TSS values between 0.36 and 0.63, and CBI values between 0.51 and 0.94.

### 3.3 Species‐ and Region‐Specific Drivers of Malaria Vector Suitability

Understanding the environmental drivers of malaria transmission is crucial for pre‐empting outbreaks. Therefore, we explored the contribution of different climatic and land‐use variables controlling the distribution of mosquito species carrying malaria. Out of six predictor variables (two of which are related to temperature, three to precipitation, and the categorical land use variable), annual precipitation stood out as the first or second most important variable for four out of six investigated species (Table 1). Annual mean temperature is also a key predictor for *An. nili* s.l., *An. coluzzii*, and *An. gambiae*, which are also the three species projected to go through increases in suitability, while the annual temperature range is important to explain the distribution of *An. moucheti* s.l., which is restricted to tropical areas. Land use generally contributed less than the climate variables.

**TABLE 1:** Relative variable importance for each variable and each species.

Species	Annual mean temp.	Temp. range	Annual prec.	Prec. of driest month	Prec. seasonality	Land use and cover
*An. gambiae*	0.074	0.015	**0.153**	0.020	0.023	0.055
*An. arabiensis*	0.028	**0.111**	0.061	0.056	0.083	0.064
*An. funestus*	0.013	0.032	**0.092**	0.033	0.047	0.075
*An. moucheti s.l*.	0.010	**0.080**	0.066	0.028	0.035	0.012
*An. coluzzii*	**0.119**	0.056	0.109	0.024	0.041	0.036
*An. nili s.l*.	**0.076**	0.028	0.066	0.010	0.040	0.069

*Note:* The importance is given as the mean absolute Shapley value, which is the average size of the contribution of each variable to the suitability estimate. Bio12 (Annual Precipitation) is an important variable across all species, while bio1 (Annual Mean Temperature) is important for several species, including *An. gambiae*. The bioclimatic variable of highest importance for the species is highlighted in bold. Variables are (from left to right): bio1 (Annual Mean Temperature); bio7 (Temperature Annual Range); bio12 (Annual Precipitation); bio14 (Precipitation of Driest Month); bio15 (Precipitation Seasonality); lulc (Land Use and Land Cover).

**FIGURE 3:**
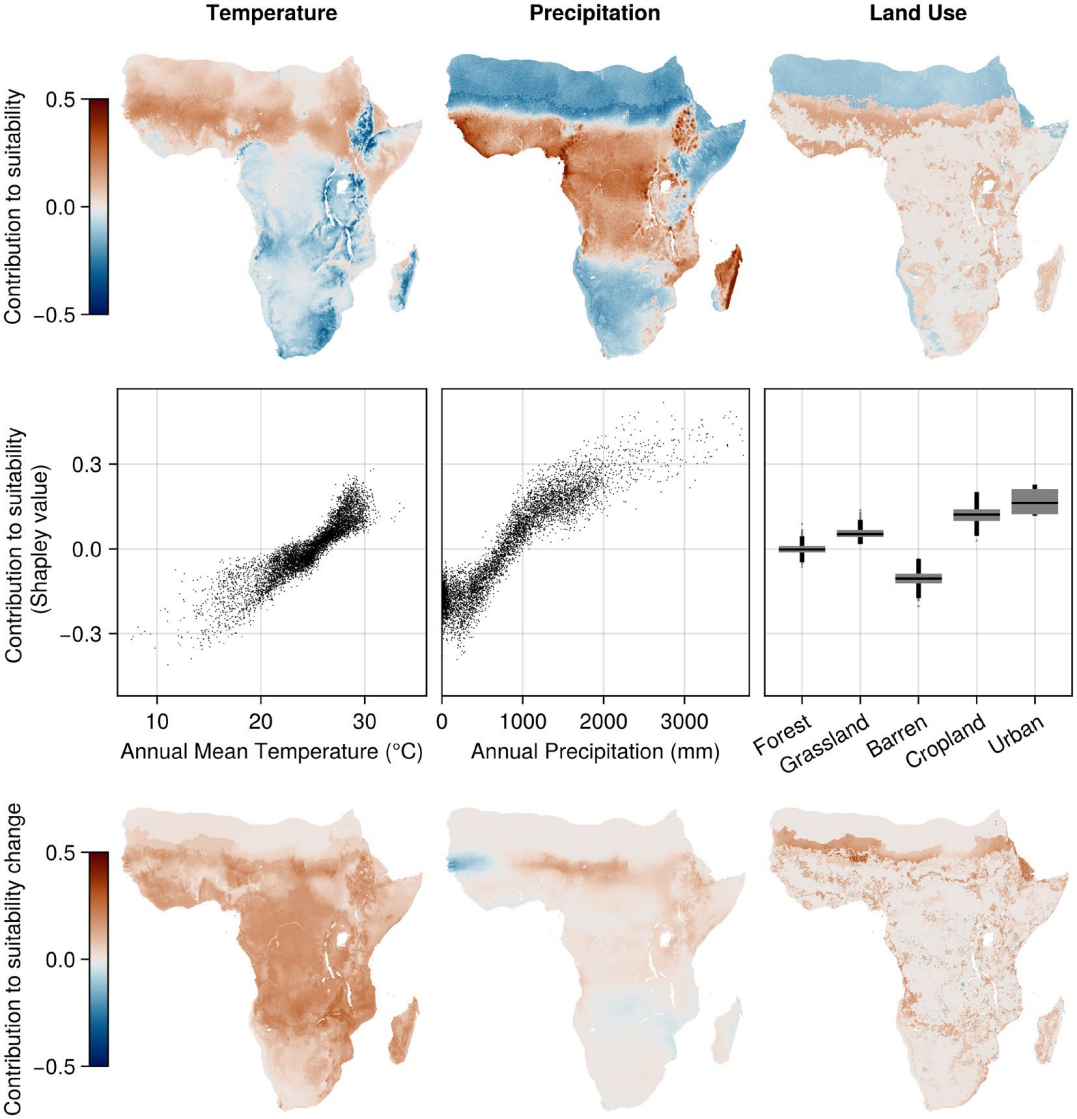
Model explanations for *Anopheles gambiae* based on Shapley values. A Shapley value measures the contribution of each variable to the final prediction for any grid cell (positive values (red)) indicate the variable increased the suitability, whereas negative values indicate the variable decreased suitability, and values close to 0 indicate no contribution whatsoever. For clarity, Shapley values for temperature variables (left row) and precipitation (middle row) variables are shown combined. Top row: Shapley values plotted for the whole region of interest. In arid areas such as the Sahara, precipitation variables contribute negatively to suitability of *An. gambiae*. Middle row: Shapley values plotted against temperature, precipitation, and land use values for ten thousand randomly drawn grid cells. Higher temperature and precipitation correlate with higher Shapley values. Bottom row: Contributions of each set of variables to the change in suitability between current and future (SSP3‐7.0, 2071–2100) conditions, demonstrate that changing temperature explains most of the increase in suitability.

Our results also reveal spatial patterns of variable contributions. For *An. gambiae*, precipitation variables explain the low suitability of this species in the Namibian, Somalian, and Sahara Desert (Figure 3), with higher precipitation linked to higher suitability. Warmer areas, such as in West Africa, have higher Shapley values for temperature variables, indicating that temperatures contribute to higher suitability of *An. gambiae*. “Cropland” and “Urban” land cover were consistently associated with higher suitability and Barren land with lower suitability, although land cover was a much less important predictor for *An. gambiae* suitability than climatic variables. The increasing suitability under future (SSP3‐7.0, 2071–2100) conditions compared to current conditions was mostly explained by temperature variables, while land use change played a role in increasing suitability at the northern edge of the range, and minor contributions from changing precipitation across the continent (Figure 3, bottom row).

Variable contributions for the other malaria vector species modelled had different geographic and environmental patterns (Figures S4–S8). For *An. arabiensis*, precipitation variables, but not temperature variables favor suitability in arid areas (Figure S4). For *An. moucheti* s.l., both temperature and precipitation variables explain the suitability in Central Africa (Figure S6).

### 3.4 Implications of Changing Mosquito Distributions

**FIGURE 4:**
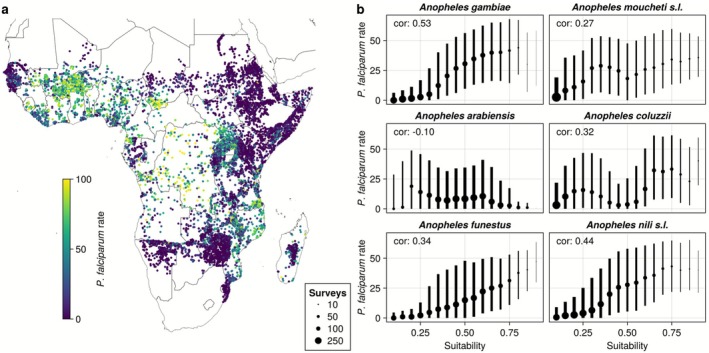
Suitability of malaria vectors is correlated with childhood malaria across Sub‐Saharan Africa. (a) Plotted survey data of childhood malaria prevalence (age‐corrected P. falciparum prevalence in children aged 2–10) show that malaria prevalence is high in Western and Central Africa. (b) Higher suitability of *An. gambiae*, *An. nili* s.l., and *An. funestus* is consistently associated with higher P. *falciparum* rates, as shown by median (dots) and interquartile ranges (lines) in each bin, as well as by the correlation coefficient. For each species, surveys were divided into overlapping bins 0.2 points wide and spaced 0.05 points apart. The size of dots represents the number of surveys in each bin.

Modelled vector abundance has been successfully used to guide malaria interventions (Larsen et al. 2020), but the usage of vector suitability maps such as those presented here to make inferences about actual malaria risk, is not necessarily straightforward, as many additional factors play a role in determining malaria prevalence, and the link between environmental suitability and mosquito abundance remains uncertain (Weber et al. 2017). To investigate the connection between vector environmental suitability and actual malaria prevalence, we analyzed if vector suitability is associated with observed prevalence of childhood malaria despite these limitations. For *An. gambiae*, *An. nili* s.l., and *An. funestus*, areas with higher suitability consistently had higher observed malaria prevalence, with the highest correlation coefficient for *An. gambiae* (*r* = 0.53) (Figure 4). The association between suitability and malaria prevalence was more unclear for the other species. The differences between species might reflect the different roles these vectors play in malaria transmission, while the overall positive correlation suggests vector suitability is a useful indicator for malaria risk.

**FIGURE 5:**
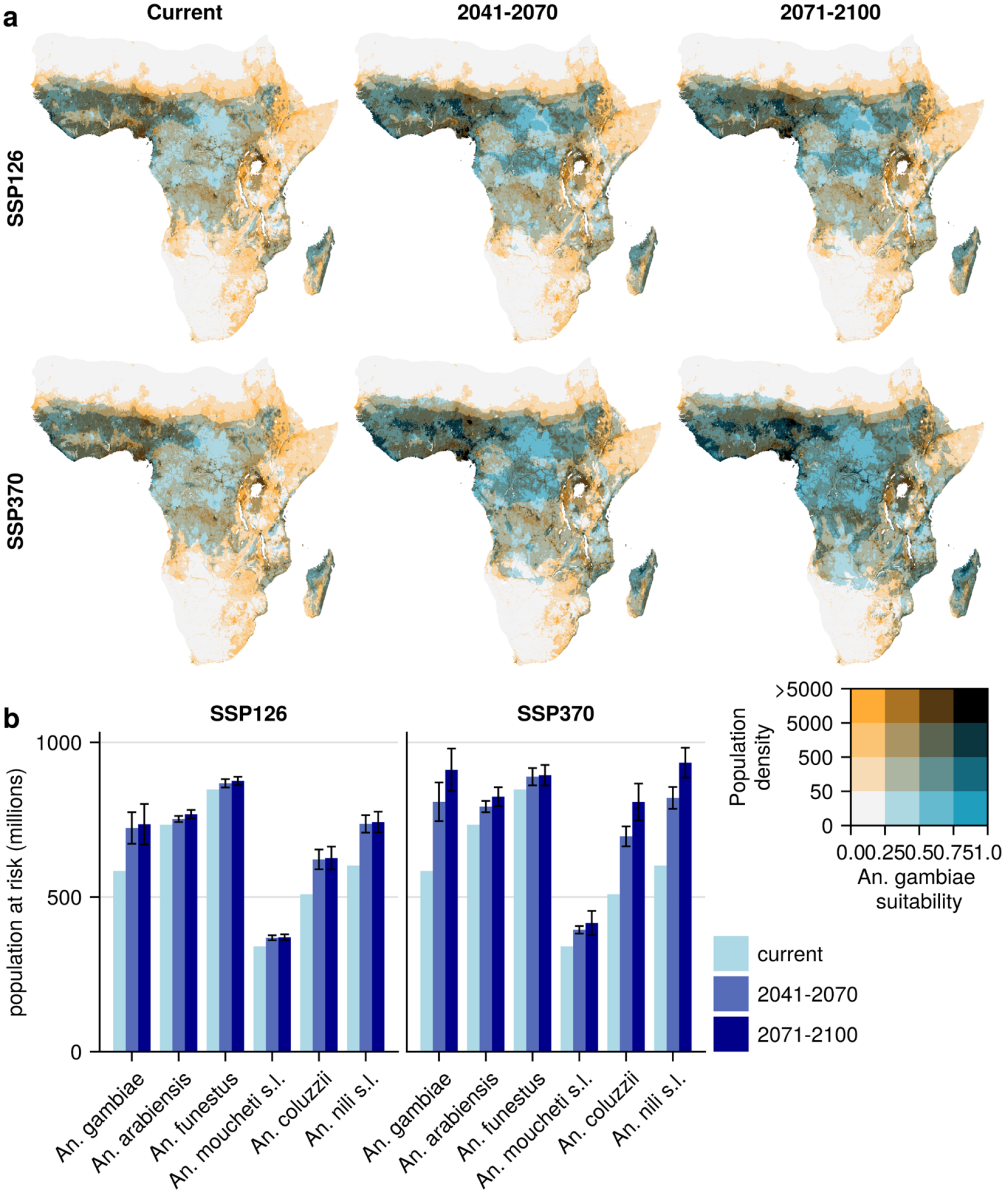
Overlaps of high climatic suitability for *Anopheles* mosquitoes and human population densities. Estimates are illustrated under current environmental conditions and future conditions, according to the low‐emission SSP1‐2.6 and high‐emission SSP3‐7.0 scenario. (a) Human population density plotted with the suitability of *An. gambiae*, the principal malaria vector, under current (left panel) and future (middle and right panels) climate scenarios. (b) Population that lives in areas highly suitable for each of six malaria vector species. Error bars indicate the standard deviation across 5 climate models used, while the bars correspond to the mean estimate.

As climate and land use change continue to reshape malaria risk, identifying new emerging hotspots where high vector suitability and human population density overlap is crucial, especially as it could mean exposing vulnerable, immunologically naive populations to threats from malaria. Currently, most areas that are both densely populated and highly environmentally suitable for *An. gambiae* are found in West Africa (Figure 5a). In the future, these areas will become even more suitable due to environmental changes. In addition, new hotspots with high human population density and high *An. gambiae* suitability will emerge in East and Central Africa, especially under the high‐emission SSP3‐7.0 scenario, underscoring the need for enhanced surveillance and control measures in these regions. Areas with increasing suitability of *An. coluzzii* and *An. nili* s.l. also overlap with densely populated areas (Figures S9–S13).

More than 800 million people currently live in areas predicted as highly suitable for *An. funestus*, the highest number for any of the investigated vectors (Figure 5b). However, while the number of people that live in areas highly suitable for *An. funestus* as well as *An. moucheti* s.l. and *An. arabiensis* will change relatively little until the end of the century, the number of people that live in areas highly suitable for *An. gambiae, An. coluzzii*, and *An. nili* s.l. will increase significantly. Under the SSP3‐7.0 scenario, the number of people living in areas highly suitable for *An. nili* s.l. and *An. gambiae* will increase to 934 million (range: 891–994 million) and 911 million (range: 852–1007 million) people by the end of the century, respectively. When also accounting for demographic changes within each country, these numbers increase to 3.5 billion (range: 3.3–3.7 billion) people for *An. nili* s.l. and 3.4 billion (range: 3.2–3.7 billion) people for *An. gambiae* under the same scenario, up to a billion people more than in a hypothetical scenario with demographic change but no future climate change (Figure S14).

While the expansion of malaria vectors continues throughout the century under the high‐emission SSP3‐7.0 scenario, in the low‐emission SSP1‐2.6 scenario, there is almost no change between the middle and end of the century (Figure 5). This highlights that aggressive climate mitigation can still stave off at least some of the effects of climate change.

## 4 Discussion

This study provides the first continental‐scale assessment of how climate and land use change may reshape human exposure to Africa's dominant malaria vector species. By modeling six dominant, widely spread malaria vectors, we find that environmental suitability for *An. gambiae*, *An. coluzzii*, and *An. nili s.l*. is projected to expand substantially over the coming decades—particularly in East and Central Africa—while remaining high in West Africa. By overlaying these shifts in suitability with current population distributions, we estimate that around 200 million additional people could be living in areas highly suitable for these vectors by the end of the century (Figure 5). Because these estimates do not account for future population growth, they represent a conservative lower bound. After accounting for demographic changes, this estimate increases to a billion people under a scenario with high population growth and emissions. These findings highlight the urgent need to adapt vector control strategies to shifting vector distributions driven by climate change, ensuring interventions remain effective in regions where malaria risk is likely to intensify.

The predicted changes in environmental suitability of malaria vectors could present a challenge to malaria control. If increasing environmental suitability would trigger higher mosquito abundance (Weber et al. 2017), this directly increases the force of infection of malaria (Lindblade et al. 2000; Mandal et al. 2011; Meyrowitsch et al. 2011). Moreover, if malaria vectors emerge in new areas, this can challenge existing control interventions that do not target the newly emerging species, for instance because the emerging species has different biting behavior, breeds in different areas, or has different seasonal population dynamics (Carnevale and Manguin 2021; Derua et al. 2012; Kitau et al. 2012; Máquina et al. 2024). The (re‐)emergence of vectors could especially present challenges in areas where malaria transmission is currently low, including in East Africa. For example, a large resurgence of malaria in Rwanda in 2017 has been linked to an increased number of *An. gambiae* mosquitoes and above‐average temperatures (Hennessee et al. 2024). While continuous control programs across Africa have brought malaria prevalence down significantly already (Bhatt et al. 2015), and ambitious goals have been set for future malaria eradication (Feachem et al. 2019), the increasing environmental suitability of key malaria vectors could challenge their continued effectiveness.

Our projections of increasing environmental suitability for key malaria vectors (Figure 2) align with observations of historical range shifts of *Anopheles* species. A recent analysis of the historical occurrence records of 21 *Anopheles* species showed that these have been recorded at increasingly polewards and upslope locations in the course of the 20th century (Carlson et al. 2023). However, since factors such as vector control efforts and the increasing human footprint (Skinner et al. 2023) may also contribute to shifts in vector distribution and malaria transmission, it is difficult to attribute any particular change to climate change (Hay et al. 2002; Snow et al. 2017). As this study highlights, mosquito vector range shifts can have major implications for human health across sub‐Saharan Africa. Therefore, there is a continued need for longitudinal field studies that can detect the changing distribution of malaria vectors to understand how these processes play out locally and globally.

Our finding that *Anopheles* vector mosquitoes are favored by climate and land use change is consistent with a recent SDM study on *Anopheles* mosquitoes (Nie et al. 2024) and our current‐day projections generally align well with the distribution maps generated by the Malaria Atlas Project (Sinka et al. 2010; Wiebe et al. 2017). However, projections in earlier studies, particularly on *An. gambiae*, predicted little change or decreasing suitability, in contrast to our results (Drake and Beier 2014; Peterson 2009; Tonnang et al. 2010, 2014). This might reflect advancements in data availability and SDM methods since these studies were published over 10 years ago, most notably the datasets published by Snow et al. and as part of the Malaria Atlas Project, both of which we use here, as the first study to do so (Massey et al. 2017; Snow 2017a). Compared to distribution maps published by the Malaria Atlas Project, our models predict lower suitability for *An. nili* s.l. in southeastern Africa, and higher suitability for *An. gambiae* in the eastern Sahel region, including South Sudan and western Ethiopia (Sinka et al. 2010). Although there are no *An. gambiae* occurrence records from this area in our dataset, a recent survey that is not in our dataset recorded the presence of *An. gambiae* in South Sudan, suggesting suitable conditions there (Petrarca et al. 2000). This illustrates remaining issues with sampling bias and sparsely sampled regions, where more research is needed to ascertain the actual distributions of malaria vectors.

Mechanistic models have been instrumental in advancing our understanding of malaria transmission by providing valuable insights into the thermal limits of parasite development and vector survival (Caminade et al. 2014; Couper et al. 2024; Murdock et al. 2016; Ryan et al. 2015; Shocket et al. 2024; Suh et al. 2024; Villena et al. 2022). Mordecai et al. (2013) influential paper on malaria transmission by *An. gambiae* concludes that malaria transmission peaks around 25°C (Mordecai et al. 2013), in part due to decreased mosquito survival and reproduction above this temperature (Agyekum et al. 2022; Gilioli and Mariani 2011; Kirby and Lindsay 2009). Numerous follow‐up studies predicted a decrease in malaria transmission across most of sub‐Saharan Africa, and especially in West Africa (Caminade et al. 2014; Colón‐González et al. 2021; Mordecai et al. 2020; Ryan et al. 2015, 2020). However, this stands in contrast with our findings that the environmental suitability of malaria vectors including *An. gambiae* will increase across sub‐Saharan Africa with climate change.

Possible explanations for this discrepancy reflect the inherent limitations of the respective modeling approaches used. In mechanistic modeling studies, it is hard to account for behavioral adaptations that allow species to survive in adverse conditions (Kearney et al. 2009). *Anopheles* mosquitoes have evolved a number of behavioral adaptations to persist in adverse conditions, including aestivation during the dry season, seasonal recolonization, and resting and feeding indoors (Lehmann et al. 2010; Mwima et al. 2023). Genetic plasticity may also play a role in adaptation to local conditions (Fouet et al. 2012; Hidalgo et al. 2018). On the other hand, regardless of adaptations, all species must have an upper thermal limit, even if it cannot be observed from current distribution patterns (Kearney et al. 2010). The uncertainty connected with extrapolation to novel climates is a crucial limitation of SDMs, particularly when no upper limit can be detected from occurrence data.

We did not find a clear upper thermal limit for several species including *An. gambiae*, which is consistent with another recent study that used SDMs to estimate the thermal limits of 7 mosquito species failed to find upper limits for five of those, including *An. gambiae* (Athni et al. 2024). This increases the uncertainty of predictions of mosquito distributions towards future climates.

We also show that precipitation, not temperature, variables are the most important predictors for most of the investigated species (Table 1). For example, we find that *An. gambiae* is associated with higher precipitation and that precipitation limits the suitability of *An. gambiae* in the Sahel region (Figure 3). In contrast, arid conditions are suitable for its sibling species *An. arabiensis*, which is consistent with earlier knowledge about the ecology of these species (Coetzee et al. 2000; Simard et al. 2009). While the role of water availability in malaria transmission has been recognized before (Smith et al. 2020, 2024; Tompkins and Ermert 2013; Yamana et al. 2016), these studies do not link it to the ecological preferences of individual vector species. The finding that climate change affects malaria vectors in a highly species‐specific way should be considered when developing predictive models, including early warning systems. Further investigations into the species‐specific responses to variations in precipitation could be key to informing vector control strategies and improving predictive models.

*An. stephensi* is an invasive, highly anthropophilic, and urban malaria vector that is native to South Asia and spreading quickly in Sub‐Saharan (Mnzava et al. 2022; Olatunji et al. 2024; Taylor et al. 2024). It was first observed in Djibouti in 2012 and has since spread to Ethiopia, Sudan, Somalia, and Kenya (Al‐Eryani et al. 2023; Mnzava et al. 2022), where it is already involved in malaria transmission (Emiru et al. 2023). We excluded this species from the current study, as our modeling approach (e.g., data sources, definition of the region of interest) was tailored to species native to Sub‐Saharan Africa and any projections for *An. stephensi* would therefore not be directly comparable to those we present here. However, multiple studies have already investigated the potential spread of *An. stephensi* under climate change scenarios, showing that this species might spread to numerous urban areas in Africa and represents yet another challenge on the path to malaria eradication (Liu et al. 2024; Sinka et al. 2020).

Recent reviews have highlighted shortcomings in SDM studies on mosquitoes (Barker and MacIsaac 2022; Lippi et al. 2023). Our study addressed some of these by using ensemble modeling, using occurrence data from the full range of species, and carefully selecting predictor variables (Araújo et al. 2019). However, several limitations remain, such as the reliance on data collected from a variety of literature sources and varying quality, the paucity of occurrence records in Central Africa, and the uncertainty associated with projecting models into no‐analogue climates (Fitzpatrick and Hargrove 2009; Veloz et al. 2012). Our study also does not capture all factors influencing mosquito distribution, such as direct human interaction, habitat modifications, or more local‐scale landscape features (Afrane et al. 2012; Lindsay et al. 2019). While we obtained acceptable to good model performance for most species, some anophelines have a very wide distribution across varied habitats and might therefore be inherently challenging to model (Goedecke et al. 2020).

Malaria remains the deadliest vector‐borne disease on earth, and any impact of climate change will have huge ramifications, in particular in sub‐Saharan Africa. In this study, we have for the first time assessed the potential implications for human health of future range shifts of the six most important African malaria vectors. We project that the suitability for three key malaria vector species will increase with climate change across sub‐Saharan Africa, putting an additional 200 million people at risk. These results highlight the need for additional research that addresses the ongoing redistribution of malaria vectors, including longitudinal field studies, experimental work on less‐studied vector species (Agyekum et al. 2021), and modeling that integrates mechanistic and correlative methods to reconcile laboratory observations of the thermal performance of *Anopheles* species with their observed distribution and observed malaria prevalence. As the world strives for malaria elimination while dealing with accelerating climate change (Feachem et al. 2019; Fornace et al. 2021; Nissan et al. 2021), we stress the need to keep ongoing control and eradication efforts efficient in the face of the anticipated distribution changes of malaria vectors to keep this target within reach.

## Author Contributions

**Tiem van der Deure:** conceptualization, data curation, formal analysis, investigation, methodology, software, visualization, writing – original draft, writing – review and editing. **David Nogués‐Bravo:** investigation, supervision, writing – review and editing. **Lembris Laanyuni Njotto:** conceptualization, methodology. **Anna‐Sofie Stensgaard:** conceptualization, funding acquisition, methodology, supervision, writing – review and editing.

## Conflicts of Interest

The authors declare no conflicts of interest.

## Supporting information


**Data S1:** gcb70610‐sup‐0001‐Supinfo.pdf.

## Data Availability

All code used in this article and instructions on how to download the data and reproduce the analysis are available on GitHub (https://github.com/tiemvanderdeure/AfricanMalariaVectors), and are archived at https://doi.org/10.5281/zenodo.17338503. Model outputs with predicted environmental suitability under current and future conditions for each species are available at https://doi.org/10.5281/zenodo.17495117. Mosquito occurrence data from Kyalo et al. (2017) and the Malaria Atlas Project are available at https://doi.org/10.7910/DVN/NQ6CUN and https://doi.org/10.5061/dryad.49p7f. CHELSA climate data is available at https://www.doi.org/10.16904/envidat.332. Land cover data is available at https://doi.org/10.5281/zenodo.4584774. Human population counts are available from WorldPop at https://hub.worldpop.org/doi/10.5258/SOTON/WP00647. Future population estimates per country are available at https://doi.org/10.5281/zenodo.14718294.
