# Supplementary material for: Climate Change Favors African Malaria Vector Mosquitoes
Source: Glob Chang Biol. 2025 Nov 26;31(11):e70610. doi: 10.1111/gcb.70610 (PMC12648371; doi:10.1111/gcb.70610)
Supplement: Supplementary file 1 — Data S1: gcb70610‐sup‐0001‐Supinfo.pdf. [file GCB-31-e70610-s001.pdf]

Supplementary Materials for

Climate change favors African malaria vector mosquitoes

Tiem van der Deure[1,2*], David Nogués-Bravo[2], Lembris Laanyuni Njotto[3,4], Anna-Sofie Stensgaard[1]

1 Section for Parasitology and Pathobiology, Department for Veterinary and Animal Sciences, University of Copenhagen

2 Center for Macroecology, Evolution and Climate, Globe Institute, University of Copenhagen

3 College of Information and Communication Technologies, University of Dar Es Salaam, Tanzania

4 Department of Mathematics and ICT, College of Business Education, Dar Es Salaam, Tanzania

* Corresponding author: Tiem van der Deure

Email: tvd@sund.ku.dk

This file contains

Figures S1-S14

Tables S1-S3

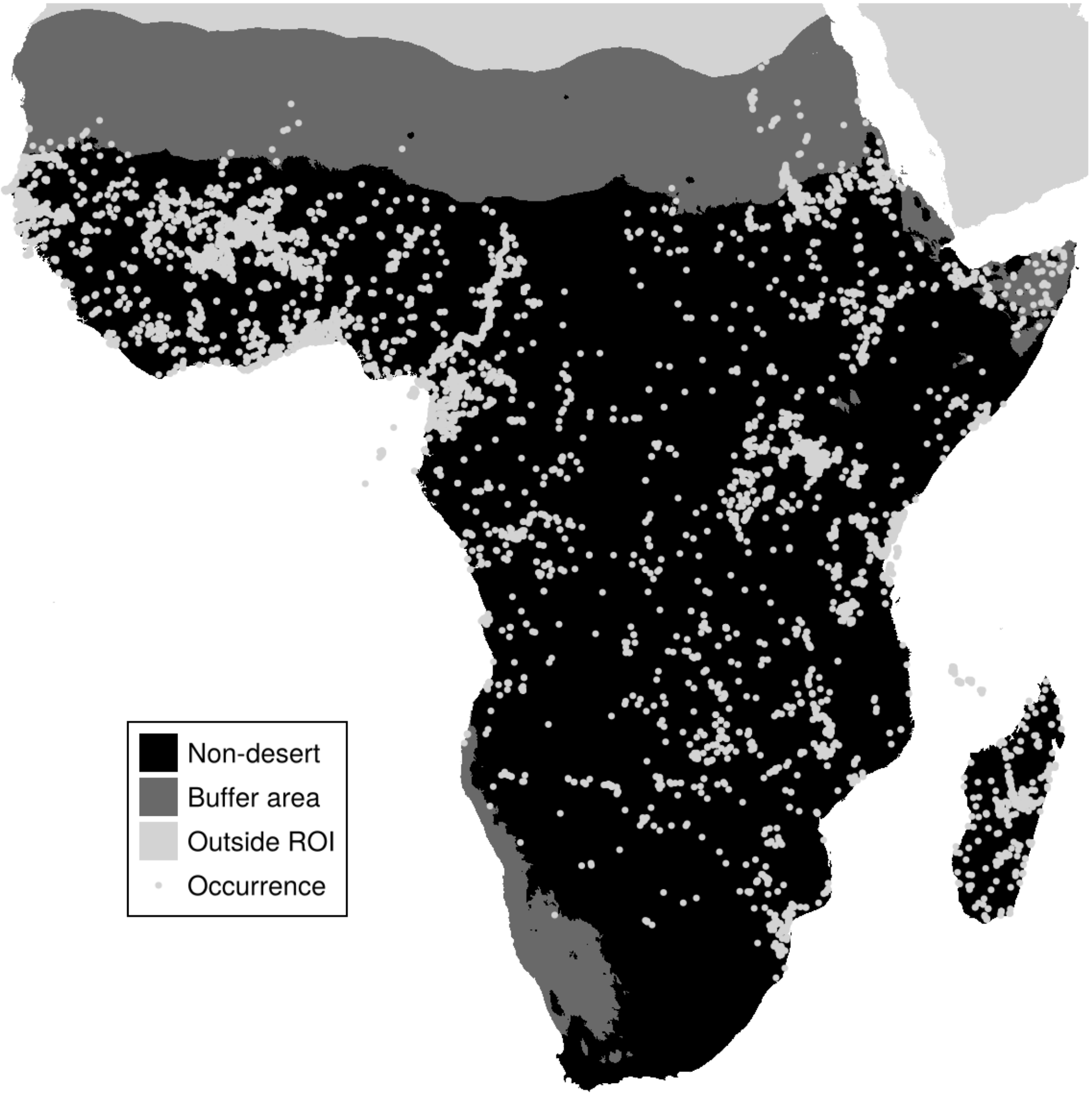

Figure S1. Region of interest used for model fitting. Background points were drawn randomly from a region of interest, which was defined by the southern boundary of the Sahara desert, buffered by 7 degrees. Non-desert areas are defined as areas with at least 200 mm precipitation per year.

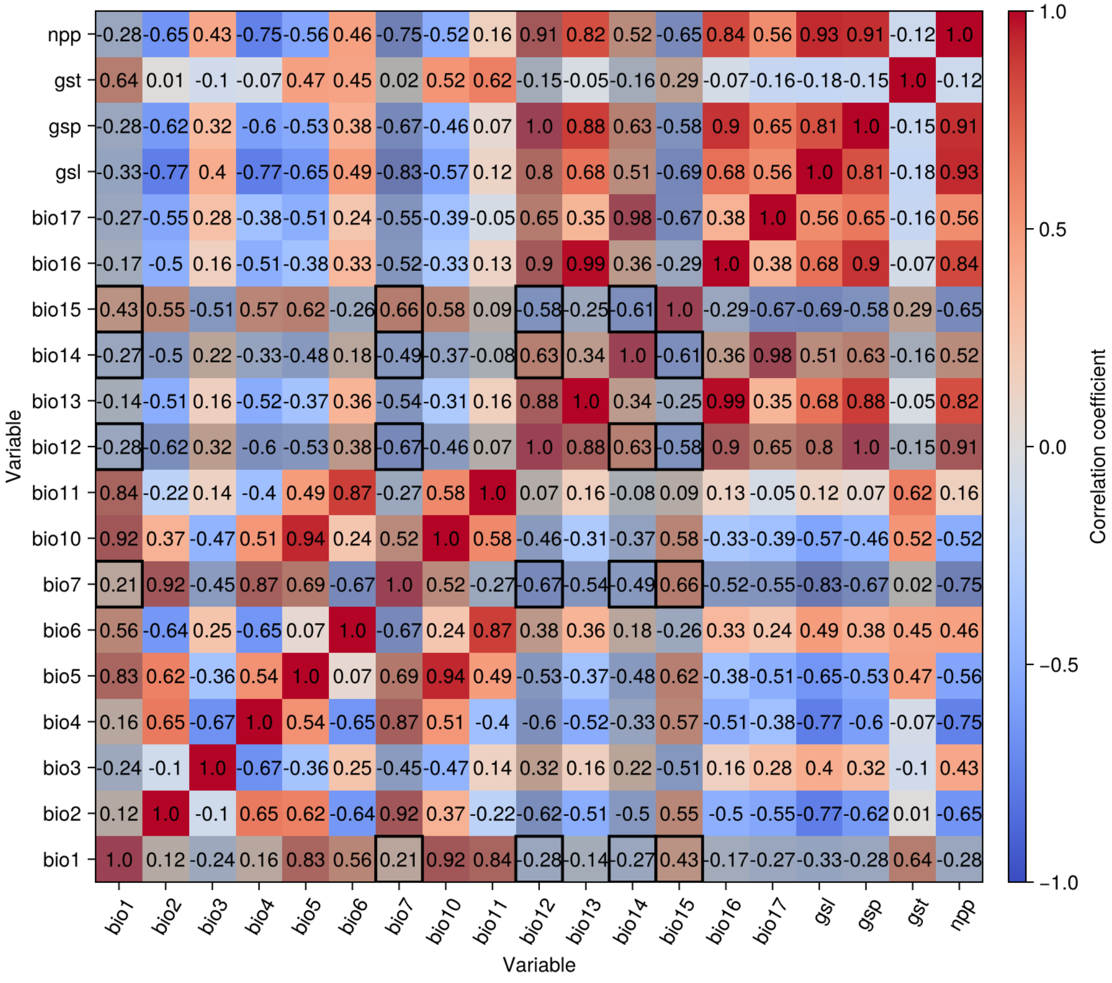

Figure S2. Correlations matrix of bioclimatic variables considered for modeling. The variables selected are highlighted. 15 out of 19 bioclimatic variables were considered as well as four extended bioclimatic variables. Selection was made based correlation (such that all correlations <0.7), simplicity of variables, and a balance between temperature and precipitation variables. Documentation for each variable is available on the CHELSA climate website: chelsa-climate.org/exchelsa-extended-bioclim/

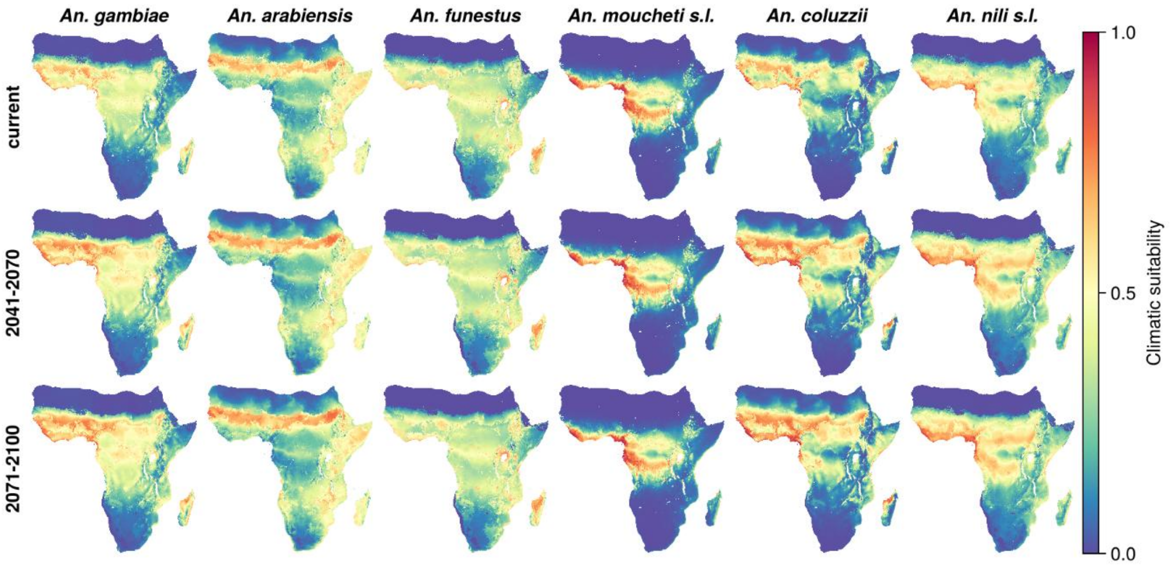

Figure S3. Climatic suitability of African malaria vector species under current and future conditions under the SSP1-2.6 scenario with low emissions.

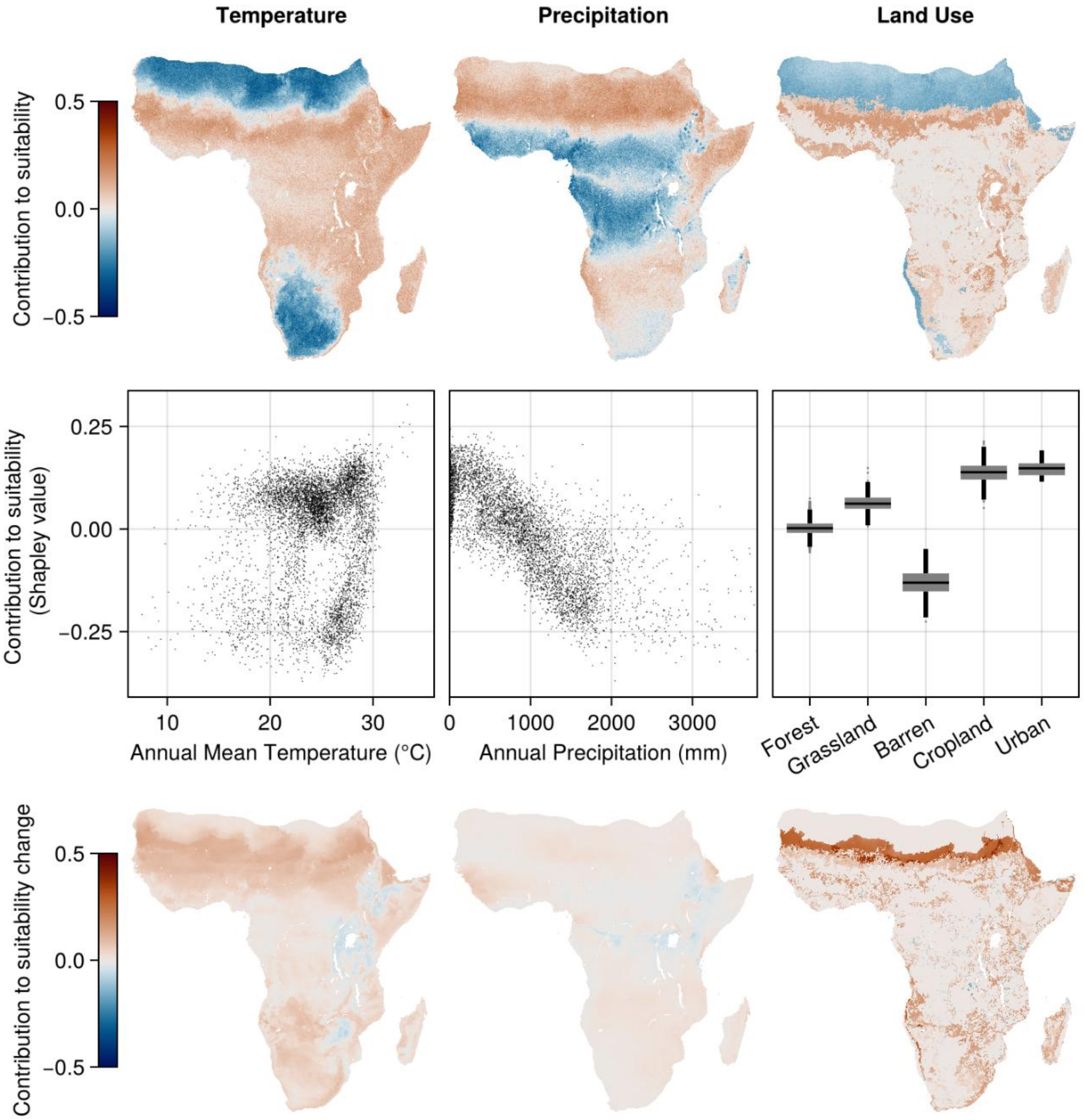

Figure S4. Model interpretations for *Anopheles arabiensis*. For interpretation of the figure, see the legend of main text Figure 3.

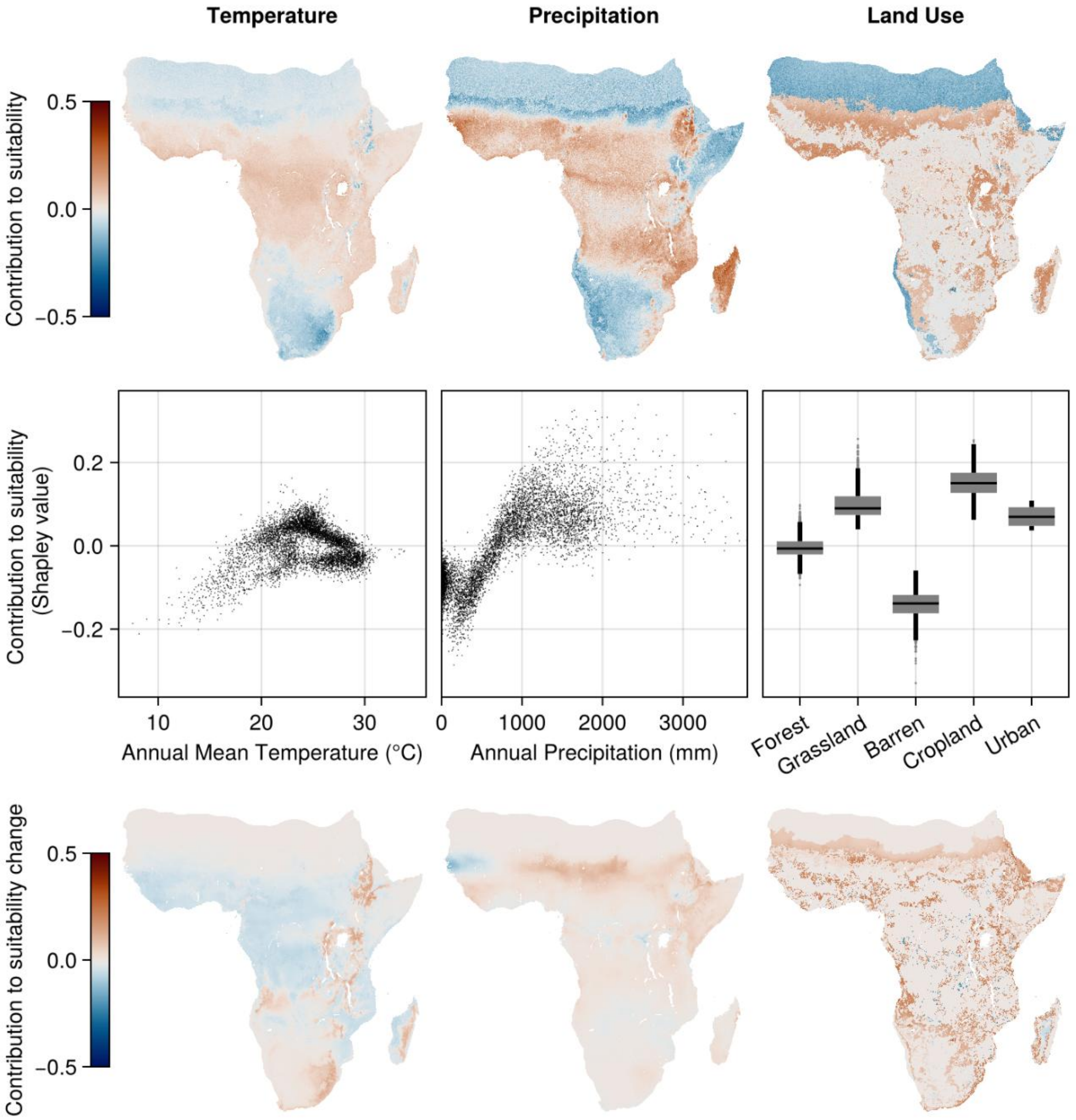

Figure S5. Model interpretations for *Anopheles funestus*. For interpretation of the figure, see the legend of main text Figure 3.

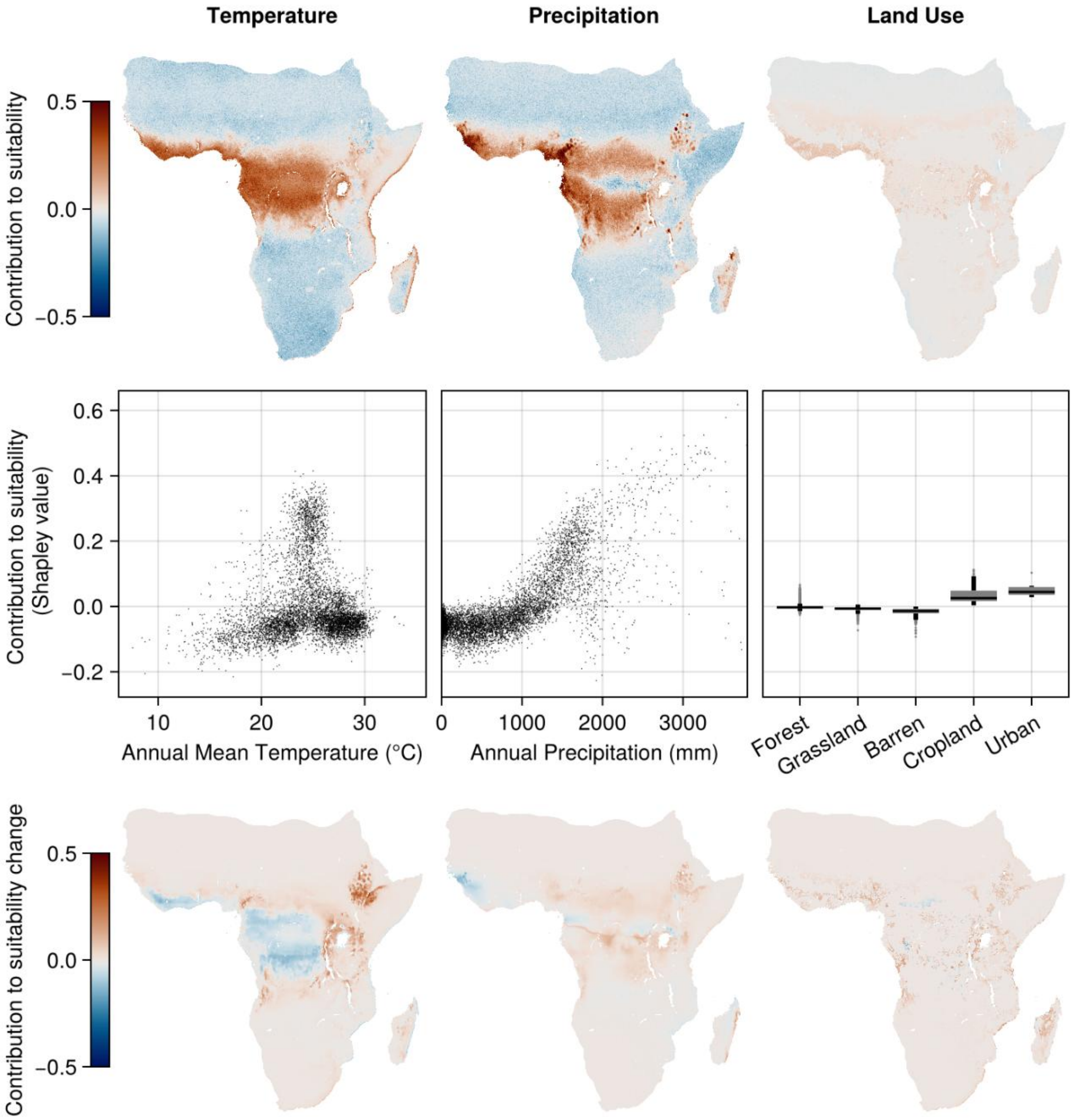

Figure S6. Model interpretations for *Anopheles moucheti s.l*. For interpretation of the figure, see the legend of main text Figure 3.

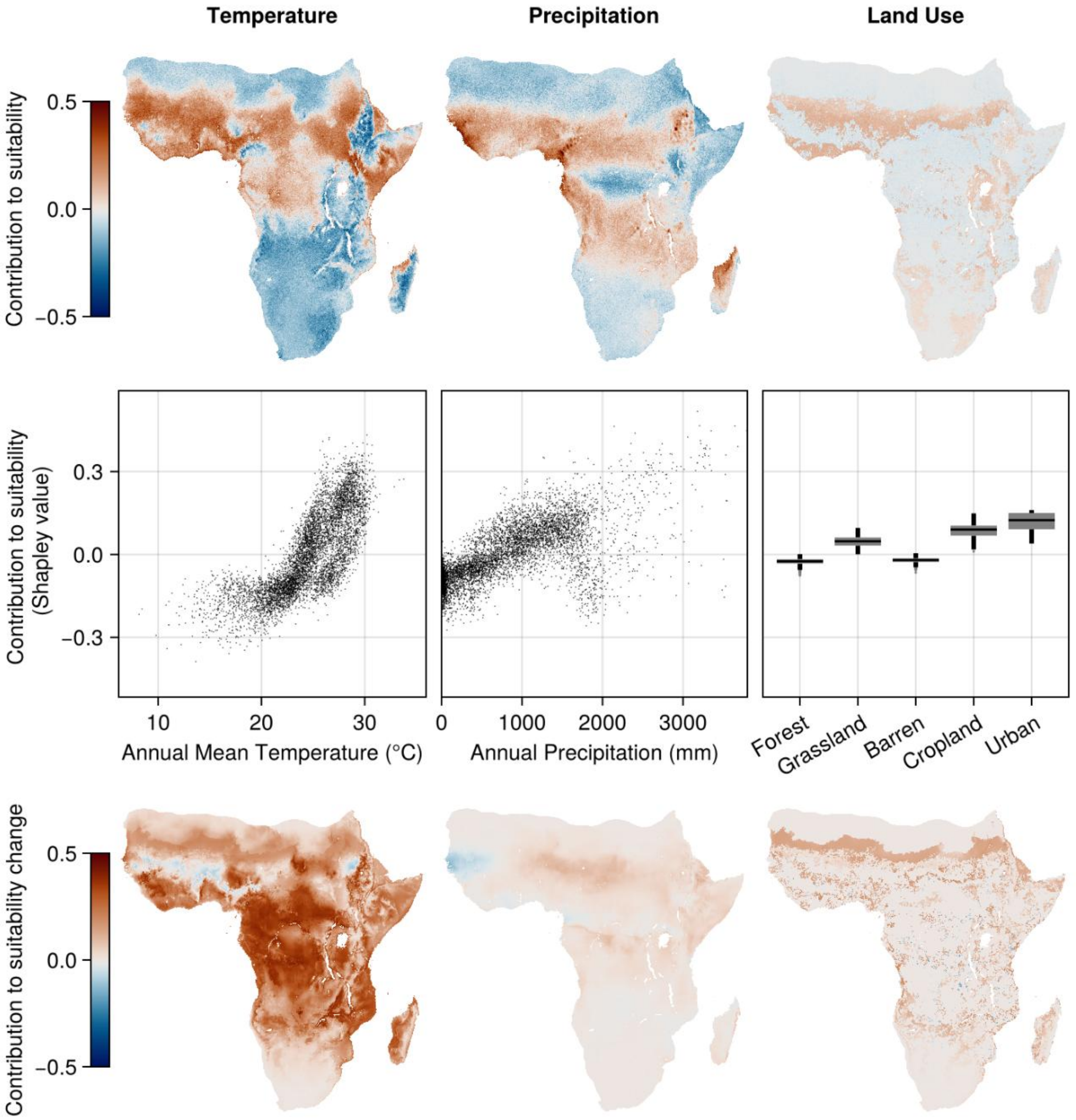

Figure S7. Model interpretations for *Anopheles coluzzii*. For interpretation of the figure, see the legend of main text Figure 3.

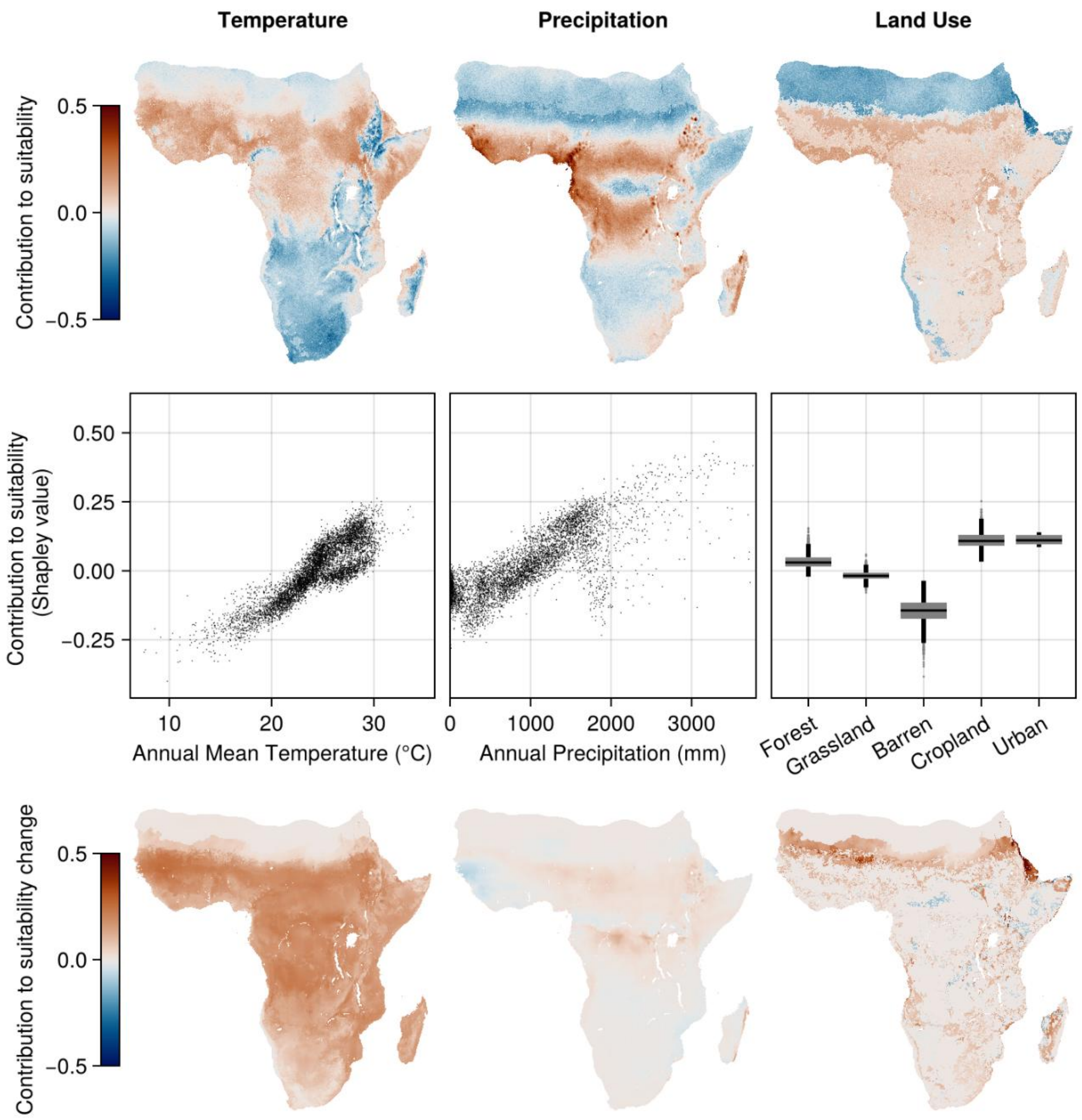

Figure S8. Model interpretations for *Anopheles nili s.l.* For interpretation of the figure, see the legend of main text Figure 3.

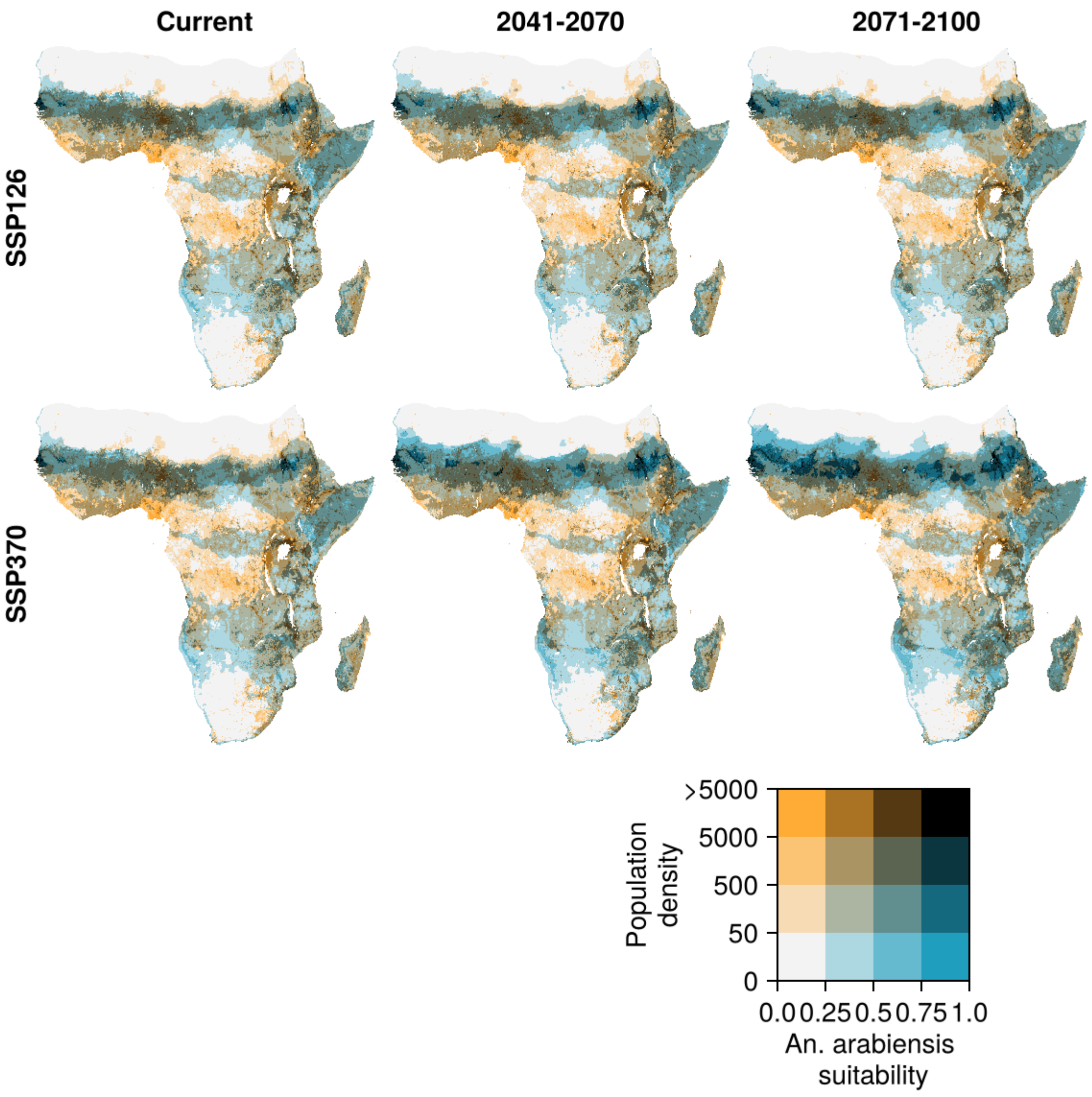

Figure S9. Overlap between human populations and areas suitable for *An. arabiensis*.

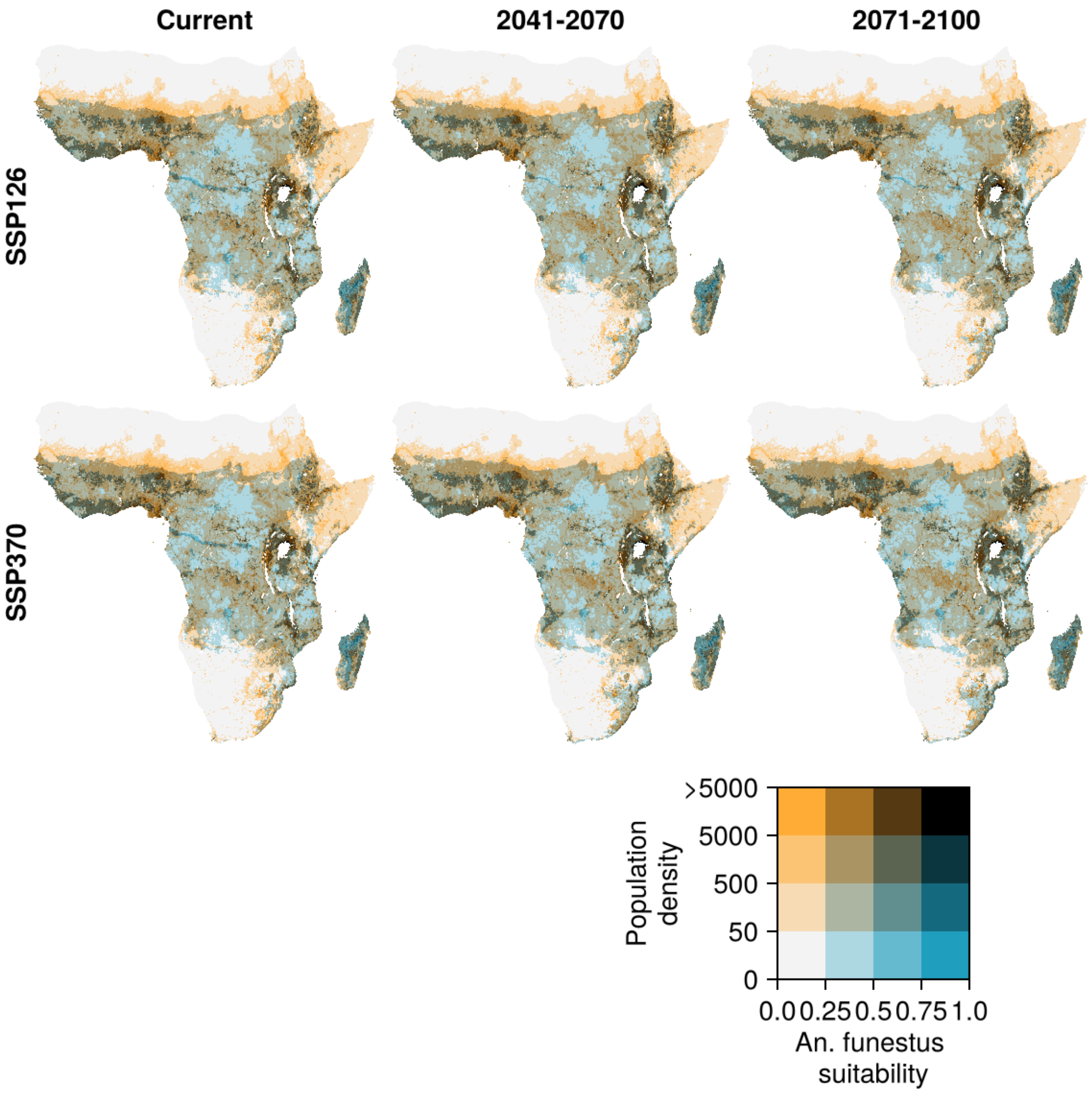

Current
2041-2070
2071-2100
SSP126
SSP370
Population density
>5000
5000
500
50
0
0.0
0.25
0.5
0.75
1.0
An. funestus suitability

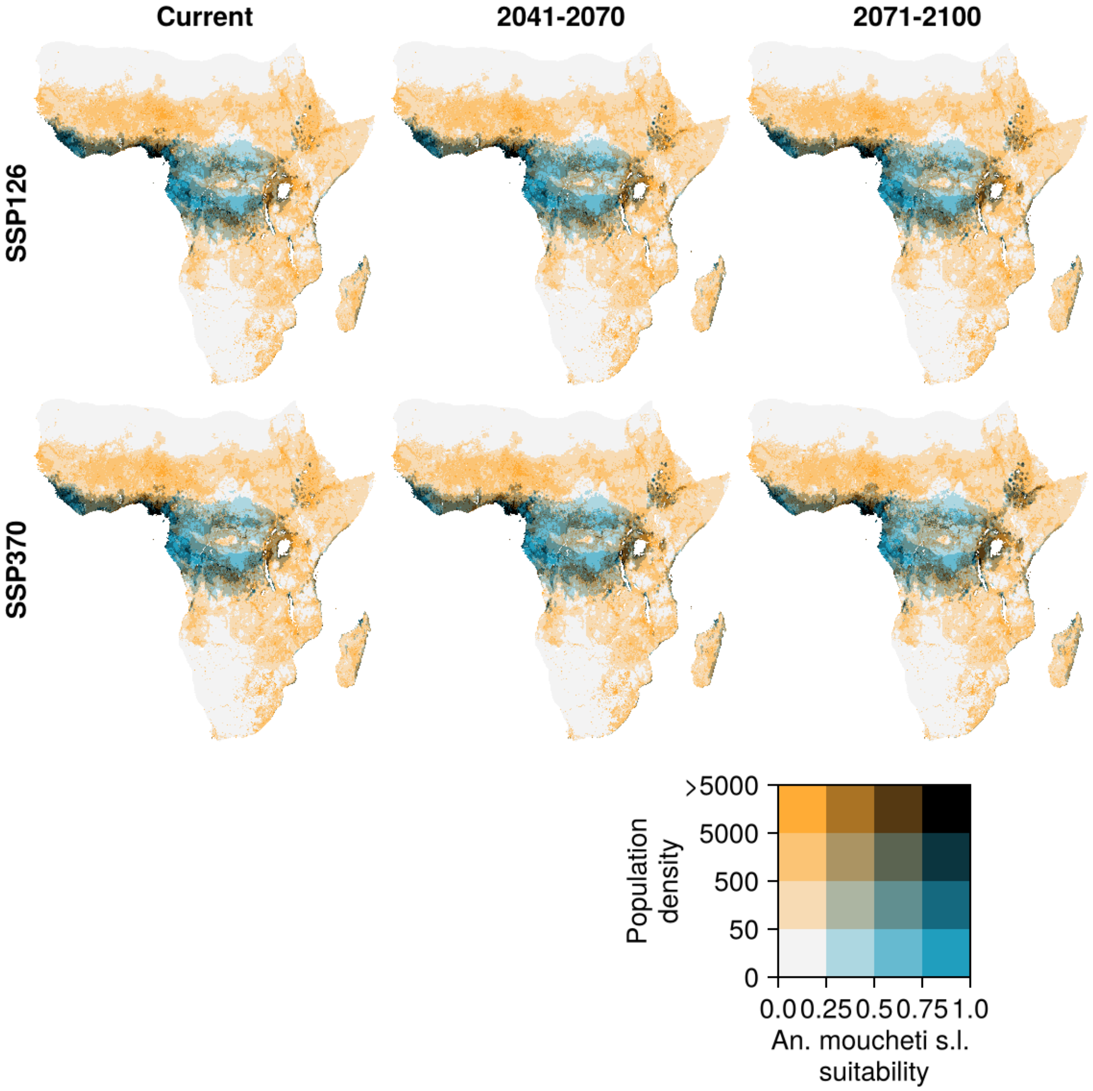

Figure S11. Overlap between human populations and areas suitable for *An. moucheti* s.l..

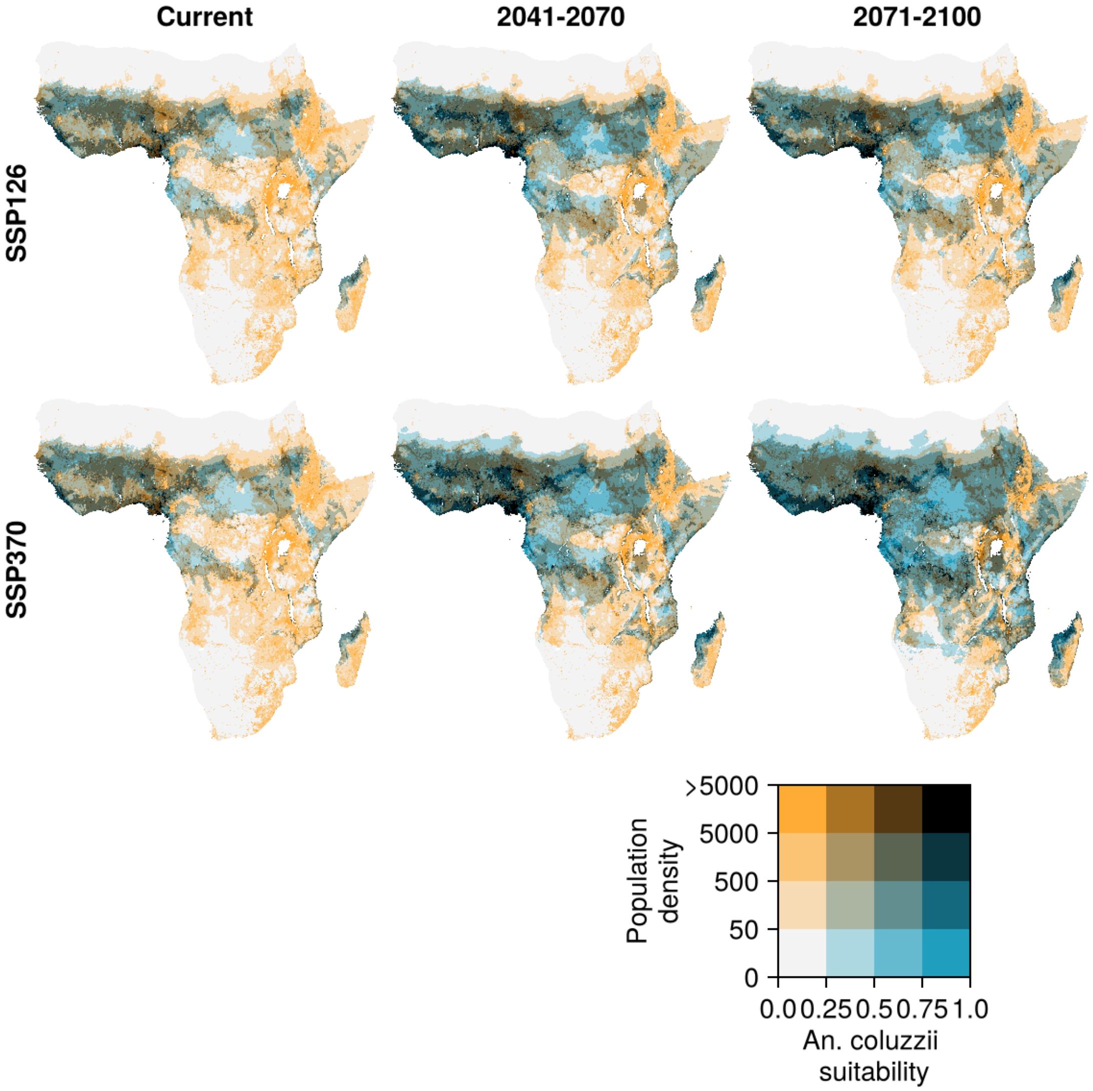

Figure S12. Overlap between human populations and areas suitable for *An. coluzzii*.

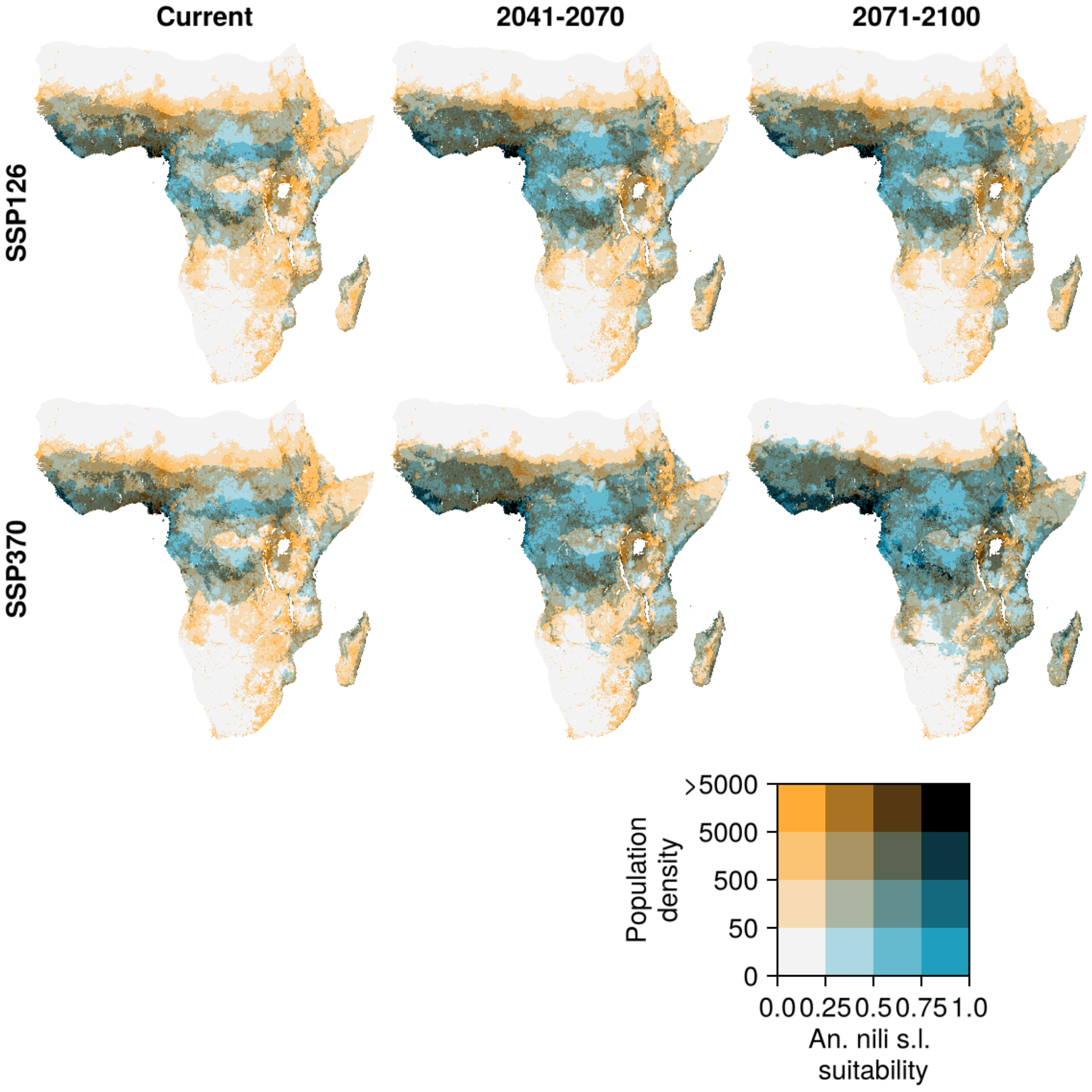

Figure S13. Overlap between human populations and areas suitable for *An. nili* s.l.

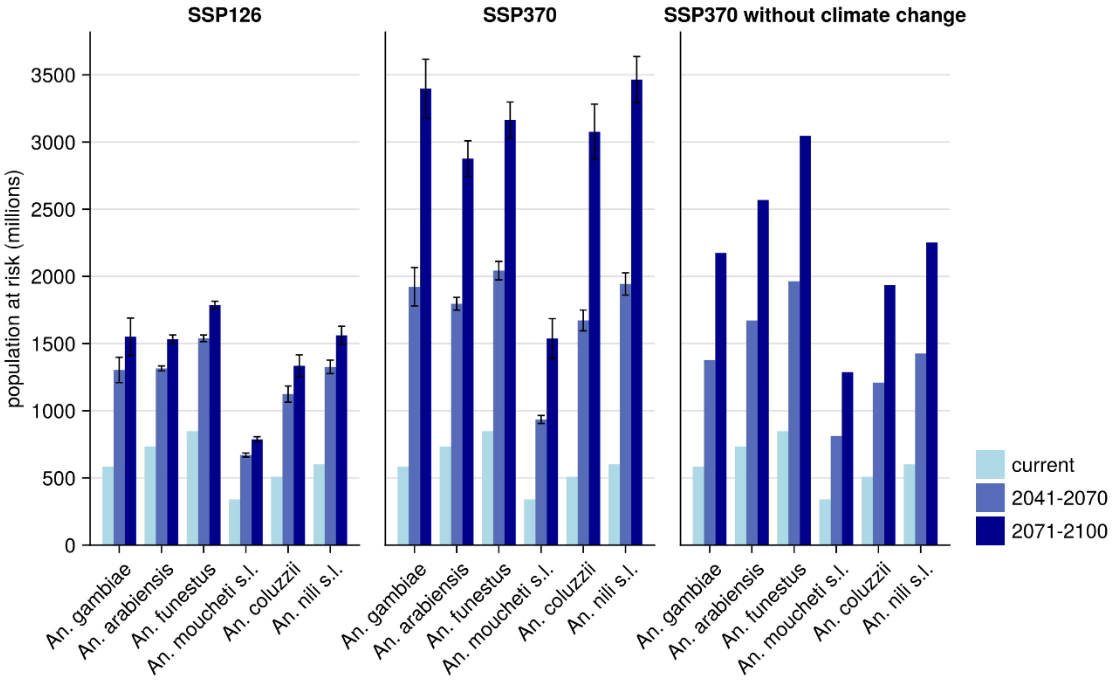

Figure S14. Estimates of the number of people living in areas highly suitable for each malaria species. The first two plots are estimates that take into account both demographic changes and changes to environmental suitability of vector species. SSP126 has both lower carbon emissions and lower population growth than SSP370. The rightmost panel shows estimates under a hypothetical scenario with population growth as SSP370, but no future climate change.

Table S1. Number of mosquito occurrences for each species after each processing step. "Unfiltered" is the total number of records without any filtering or de-duplication. "Post-1980" is the number of records remaining after removing records from sampling completed before 1980. "Pre-2010" is the number of records remaining after then removing records from sampling started after 2010. "Spatial thinning" is the number of records remaining after thinning records to a distance of 10 km (thus also any duplicate records).

Species	Unfiltered	Post-1980	Pre-2010	Spatial thinning
An. gambiae	2352	2349	2085	1290
An. arabiensis	4906	4549	4109	2773
An. funestus	1989	1959	1812	1168
An. moucheti s.l.	604	303	290	166
An. coluzzii	1732	1732	1457	888
An. nili s.l.	1023	625	584	354

Table S2. Ensemble model performance using forward variable selection. The average AUC score is given for each species, with the score for the training data in brackets. Selected predictors in the optimized set are in the third column. For definitions of each considered bioclimatic variable, see the CHELSA website: https://chelsa-climate.org/exchelsa-extended-bioclim/. lulc: Land Use and Land Cover; AUC: Area Under the receiver operating Curve.

Species	AUC	Predictors
An. gambiae	0.77 (0.78)	lulc, bio13, bio6
An. arabiensis	0.73 (0.76)	lulc, bio4, bio6, npp, bio5, bio14
An. funestus	0.76 (0.80)	lulc, gsl, bio3, bio1, bio2, bio6, bio16, gst, bio4
An. moucheti s.l.	0.85 (0.93)	lulc, bio2, bio4, npp, bio15
An. coluzzii	0.82 (0.85)	lulc, bio11, bio13, gst, gsl, bio3, bio10
An. nili s.l.	0.74 (0.80)	lulc, bio6, bio13, bio2, bio15, bio10, bio4

Table S3. Ensemble model performance using selected variables. The average testing score is given for each metric and each species, with the score for the training data in brackets. The model performance shown was calculated from the mean prediction of the ensemble. AUC: Area Under the receiver operating Curve; TSS: True Skill Statistic; CBI: Continuous Boyce Index.

Species	AUC	TSS	CBI
A. gambiae	0.76 (0.79)	0.44 (0.47)	0.88 (0.99)
A. arabiensis	0.73 (0.76)	0.36 (0.41)	0.94 (0.99)
A. funestus	0.72 (0.78)	0.38 (0.42)	0.81 (0.97)
A. moucheti s.l.	0.84 (0.92)	0.63 (0.73)	0.51 (0.96)
A. coluzzii	0.82 (0.84)	0.55 (0.56)	0.82 (0.98)
A. nili s.l.	0.72 (0.79)	0.40 (0.46)	0.70 (0.98)
